# Technological creep masks continued decline in a lobster (*Homarus gammarus*) fishery over a century

**DOI:** 10.1038/s41598-022-07293-2

**Published:** 2022-02-28

**Authors:** Alf Ring Kleiven, Sigurd Heiberg Espeland, Stian Stiansen, Kotaro Ono, Fabian Zimmermann, Esben Moland Olsen

**Affiliations:** 1 Institute of Marine Research, Bergen, Norway; 2 Department of Natural Sciences, Centre for Coastal Research, University of Agder, Kristiansand, Norway; 3 Institute of Marine Research, Nye Flødevigveien 20, 4817 His, Norway

**Keywords:** Marine biology, Time series, Environmental impact

## Abstract

Fishery-dependent data are frequently used to inform management decisions. However, inferences about stock development based on commercial data such as Catch-Per-Unit-Effort (CPUE) can be severely biased due to a phenomenon known as technological creep, where fishing technology improves over time. Here we show how trap improvement over nine decades has driven technological creep in a European lobster (*Homarus gammarus*) fishery. We combined fishing data, experimental fishing with contemporary and older trap types, and information on depletion effects during fishing seasons. The resulting standardized CPUE time series indicates a 92% decline in lobster abundance between 1928 and 2019 compared to 70% if technological creep is not corrected for. Differences are most pronounced within the last 40 years when the most substantial shift in gear technology occurred: an uncorrected CPUE index suggests an 8% increase in lobster abundance during this period, while the corrected CPUE index declined by 57%. We conclude that technological creep has masked a continuous stock decline, particularly in recent decades and largely driven by the shift from one- to two-chambered traps, as well as the ability of newer trap designs to capture larger lobsters. Our study confirms the importance of adequate standardization, including technological development, when using fishery dependent CPUE for monitoring and management of data-limited fisheries.

## Introduction

Fisheries management relies heavily on relative indices of abundance to inform stock assessment and advice^1^. However, fisheries-independent time series on the dynamics of exploited resources are typically expensive and labour-intensive, and therefore, available for only a small proportion of global fish stocks. In contrast, most global fisheries are considered data-limited^2^ and rely largely on fisheries-dependent catch and effort data. Catch-Per-Unit-Effort (CPUE) time series from fisheries are often used as a proxy for abundance when fishery-independent data are not available; they suffer, however, from a lack of standardization and are prone to bias as fisheries change over time^3,4^. Therefore, CPUE standardization^5^—the process of correcting for the impact of factors other than abundance that influence the temporal trend in CPUE—is a crucial step into deriving indices which reliably reflect the status of a stock. Such additional factors most often relate to catch efficiency and include, for example, changes in vessel size, engine power and gear type. This is especially important as fishers often improve their fishing methods over time to increase catch efficiency; this is often termed technological creep^6^. Whereas such information is often available in large-scale industrial fisheries (e.g. trawling and long-line)^6–8^, it is usually lacking in data-limited fisheries, notably small-scale coastal fisheries using gear such as trap; often with limited or no reporting requirements. This contributes to a lack of data and monitoring in many coastal systems that are among the most productive yet depleted marine ecosystems^9,10^.

The European lobster (*Homarus gammarus,* hereafter ‘lobster’) has long supported an important coastal fishery in Norway; export of live lobsters to central Europe started around 1650. Fishing regulations were initially introduced in 1849^11^ in the form of a closed season (July 15th to end of September)^12^. Even though new regulations—such as increased Minimum Landing Size (MLS) and shorter fishing season—have been implemented, the fishery has decreased to historical low levels^13^. Monitoring of the lobster fishery is based on a fishery-dependent time series (CPUE) extending back to 1928 in which annual effort and landings were estimated from a non-random selection of volunteer commercial fishers^13^. However, the CPUE time series has neither been standardized nor adjusted for technological creep and may, therefore, be highly biased. This is further exacerbated by a shortening of the fishing season that may mask local depletion effects on catch rates, as lobsters tend to be fished out in the beginning of the season, resulting in declining catch rates with time^14^. Globally, different CPUE standardization methods have been used in lobster stock monitoring^15–17^. In data-limited fisheries, CPUE standardization models must be tailored to the available data and are naturally limited by the time series at hand.

In this study, we combined two fishery-dependent 90 year-long time series (lobster CPUE and phenotypic data started in 1928 and 1921, respectively) with a desktop study on use of trap types through time, and experimental data on trap efficiency, to standardize the raw CPUE data. This enabled us to estimate technological creep in a traditional coastal fishery and provide an historic baseline index that reflects more accurately the changes that have occurred in the fishery and lobster stock. Our results revealed how technological development in lobster trap design has changed catch efficiency in Norway’s coastal lobster fishery over the last 90 years, and not correcting for these technological changes has masked continuous decline in lobster abundance over time. We go on to propose a new approach for CPUE standardization in trap fisheries.

## Results

### Mapping the trap usage over time

A total of 39 interviews were conducted to map the historical use of different trap types, indicating that four main trap types dominated the fishery at different times during the last 90 years. At the beginning of the time series, from 1928 onward, fishers mainly used one-chambered cylindrical wooden lath traps with two entrances (henceforth, cylindrical traps). From the 1950s to beginning of the 1980s one-chambered box shaped wooden traps (wooden traps) were used predominately. The use of an additional chamber to reduce potential escape of lobsters^18–20^ was first reported in the 1970s. In the 1980s, the use of these two-chambered box shaped wooden traps with only one entrance (wooden two-chamber traps) increased. Since the beginning of 2000, the use of two-chambered steel framed traps with two entrances (synthetic two-chamber traps) has predominated.

### Estimating catch efficiency of different trap types

Catch efficiency was estimated for the four main trap types using experimental fishing data. These data consisted of 326 trap hauls with 157 captured lobsters. The analysis showed that there was a significant difference in catch efficiency between the two oldest trap types and the newer trap types (Supplementary Table S1). In addition, we found a significant effect of bottom depth (right-skewed unimodal distribution) and significantly elevated catch rates within designated lobster reserves (compared to areas outside of such reserves) (Supplementary Table S1). After standardization of these effects, the mean lobster catch rate per trap was estimated at 0.22 for the old cylindrical trap, 0.28 for the old wooden trap, 0.75 for the wooden two-chamber trap, and 0.88 for the synthetic two-chamber trap. Estimated catch efficiency per trap was, thus, found to increase nearly threefold between the oldest and newest trap types. These catch efficiency estimates were used to correct fishing effort for the different trap types in the subsequent CPUE standardization.

### Size selectivity of trap types

**Figure 1:**
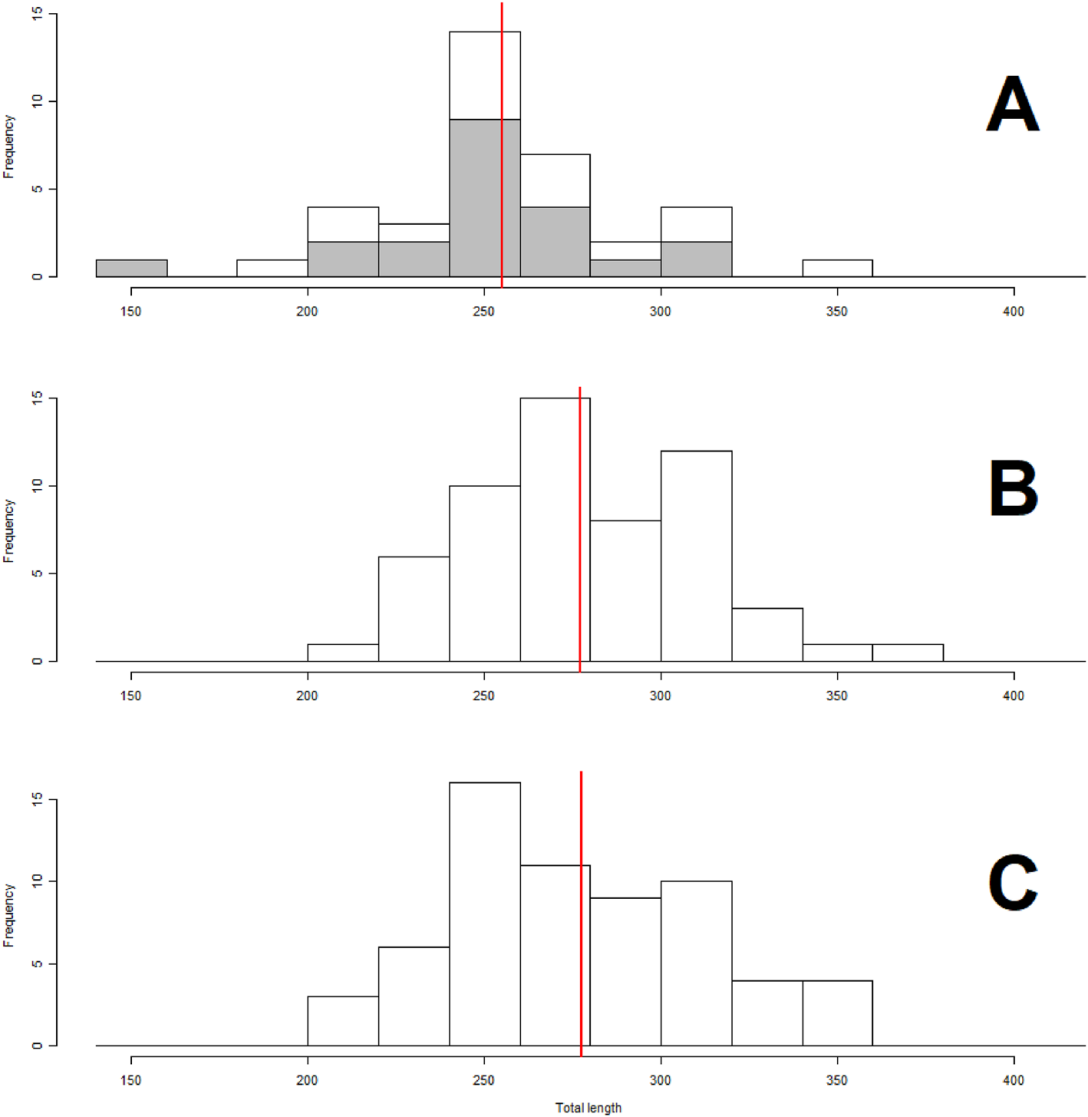
Differences in size distribution (total length measured in millimetre) between different trap types. Figures showing the number of lobsters in each size group according to the different trap designs. (**A**) Represent the two oldest designs with one chamber, the cylindrical (white) and wooden trap (grey). (**B**) Represent wooden two-chamber traps and (**C**) represent the synthetic two-chamber traps. The red vertical line is the average length of each group.

All lobsters captured in the experimental fishing were measured for total length (TL) to the nearest mm. During the experimental fishing, there was no significant difference in mean total length of lobsters captured by the two oldest trap types (cylindrical: 260 mm TL, SE = 9.9, and wooden: 251 mm TL, SE = 7.4, Fig. 1). Neither was there any significant difference between the wooden two-chamber traps (277 mm TL, SE = 4.1) and the synthetic two-chamber traps (277 mm TL, SE = 4.5). However, there was a significant difference between mean total length of lobsters caught in the two oldest (255 mm TL, 5.9 SE) vs the two newest trap types (277 mm TL, 3.3 SE) (p < 0.05).

**Figure 2:**
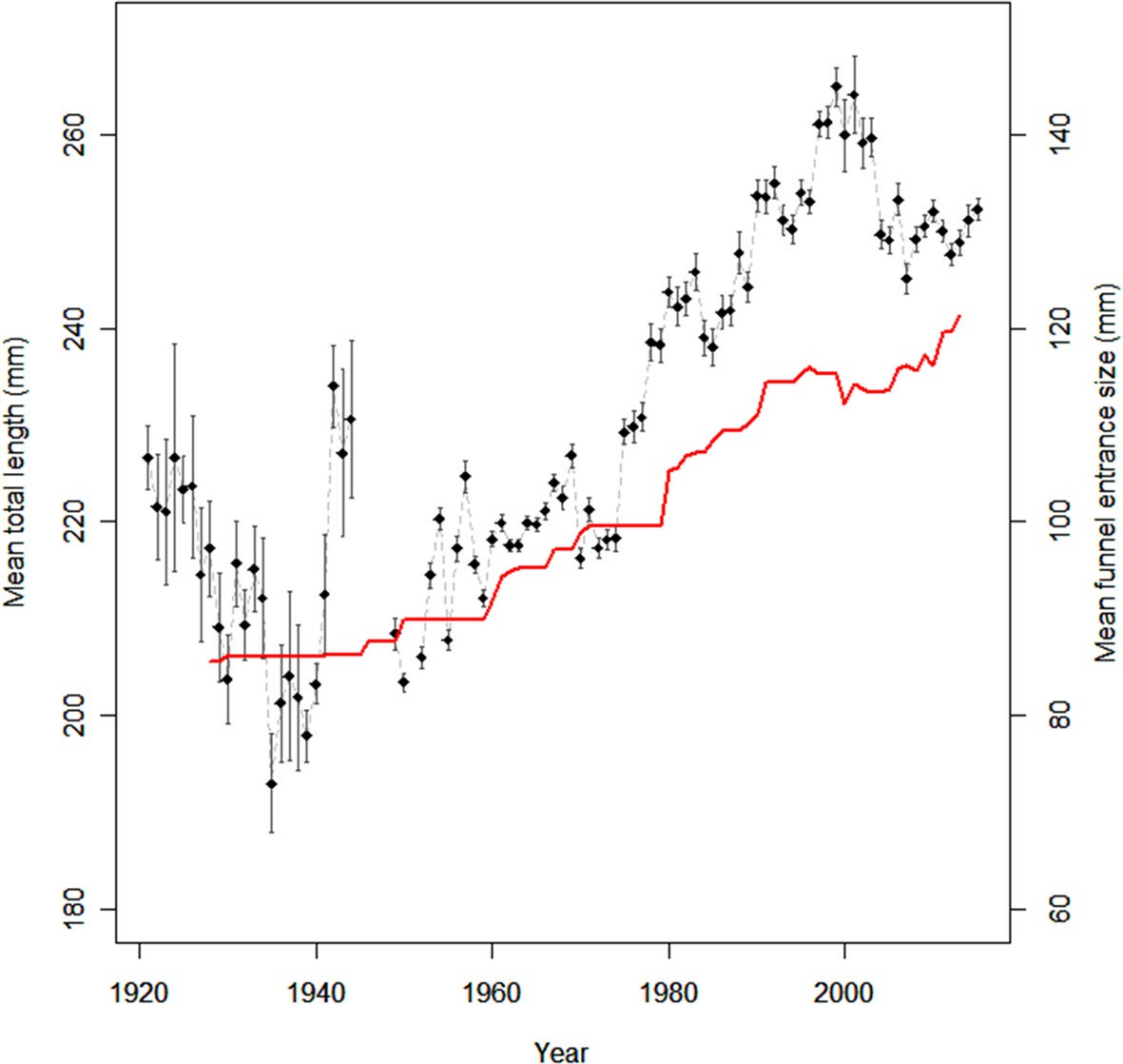
Development in average length of lobster caught in fishery from 1921 to 2015 along with an average increase in funnel size. Black points indicate yearly average length with vertical lines show 95% CI for the mean. Red line shows the estimated mean size of trap funnel eye in the time series estimated trough interview data and average funnels eye size of different traps.

Annual length data collected from fishers (1921–2019, n = 149,642) shows that mean total length of lobsters captured (released and landed) has changed throughout the time series (Fig. 2). From 1921 until 1940 the data indicate a decrease in mean total length of lobsters, followed by large variation and data gaps during the 1940s, and a substantial increase from 1949 to 2000. From 2000 to 2007, mean total length decreased and then stabilized after Minimum Landing Size (MLS) was increased in 2008.

### Time series standardization and correction for technological creep

**Figure 3:**
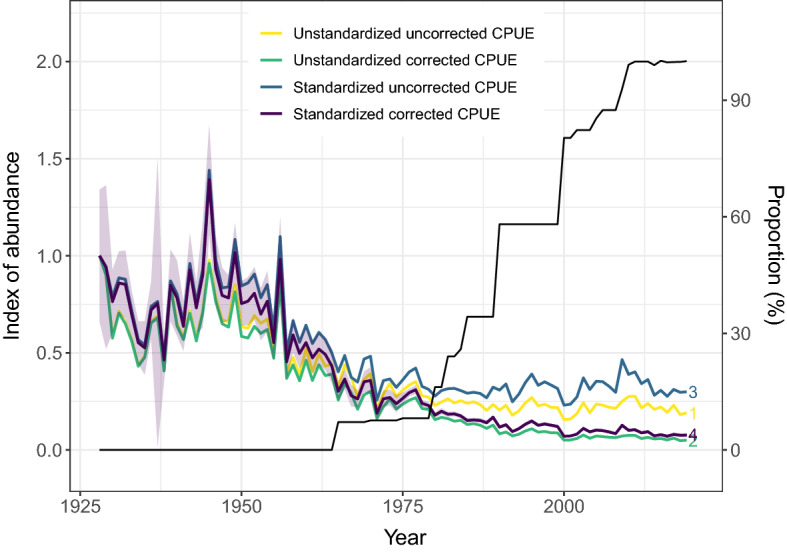
Lobster abundance indices (standardized and corrected for technological creep) based on the depletion model (purple—4) demonstrate a different trend in lobster population compared to the models that were unstandardized and uncorrected (yellow—1), unstandardized and corrected (green—2), and standardized uncorrected (blue—3). “Correction” refers to the direct adjustment of the raw CPUE data to account for technological creep while “standardization” refers to further adjustment made through statistical modelling to capture the effect of season depletion of lobster as well as other environmental effects. The light purple polygon indicates the 95% confidence interval for the depletion model (purple—4) that accounts for the trap types and number uncertainty and was derived by resampling. The black line shows the prevalence of two-chamber traps (wooden and synthetic pooled since these two had the most significant effect on the catch) and relates to the scale on the right side (proportion).

Between 1928 and the mid-1950s, the unstandardized annual catch rate (CPUE) reported by commercial fishers showed large variation and no clear trend (Fig. 3), except an increase in CPUE related to the World War II. From the mid-1950s the annual catch rate started to decrease and levelled off at a low level around 1980. The trends are consistent between unstandardized CPUE, uncorrected (for technological creep) standardized CPUE, and corrected standardized CPUE that includes information on trap type, their catch efficiency and depletion effects (Fig. 3). However, our findings indicate that both unstandardized and uncorrected standardized CPUE result in substantial overestimation of the current levels of CPUE and, thus, abundance. “Correction” refers to the direct adjustment of the raw CPUE data to account for technological creep while “standardization” refers to further adjustment made through statistical modelling to capture the effect of seasonal depletion of lobster and spatial distribution of the fishery.

Corrected standardized CPUE indicated a 92% decline between 1928 and 2019, compared to declines of 70% and 81% in uncorrected standardized CPUE and unstandardized CPUE, respectively (Supplementary Table S2). Therefore, using a standardized CPUE index in 2019 without fully correcting for trap type used and catch efficiency resulted in a 392% overestimate of population status due to unaccounted technological creep. While during the first half of the time series, uncorrected and corrected CPUE indices followed largely the same pattern, they diverged strongly during the most recent 40 years (Fig. 1, Supplementary Table S3). Specifically, correcting for technological creep suggested a decline in standardized corrected CPUE of 57% between 1980 and 2019, compared to an increase of 8% when technological creep was not accounted for.

Corrected standardized CPUE estimated by the main model (including depletion effect) and alternative model (using season length) were nearly identical (Supplementary Fig. S1). Model inspection showed that both models provide a good fit and explain the data well (Supplementary Figs. S2, S3). Year effect and depletion effect—or season length, respectively, were found to be statistically significant (Supplementary Fig. S4). The intraclass correlation of area as random intercept was 0.32, indicating that a significant proportion of the observed variation is linked to spatial differences.

## Discussion

By combining local knowledge of historic fishing gear use and experimental fishing, our study shows how trap improvement has driven significant long-term technological creep in Norway’s coastal fishery for European lobster. We find that standardized estimates of CPUE suggests a stable trend in lobster abundance in decades following the 1980s when not corrected for technological creep; while correcting for the increase in catch efficiency indicates that the CPUE index, likely in concert with abundance of the lobster stock, has declined substantially. This confirms that the introduction of two-chamber traps in the 1980s led to increased catch efficiency. The increased catch efficiency by this trap design and the fact that it captures a wider length distribution of lobsters than older trap types, have likely masked a dramatic stock decline over the last 40 years. It is in the later part of the time series that technological creep significantly masks the continuous downward trend in catch rate. While using uncorrected CPUE—both standardized and unstandardized—also indicates a decrease over the entire time series, the extent of the decline is substantially underestimated. These findings underscore the risk for large bias in determining current stock status relative to historic levels when using CPUE data not sufficiently corrected for changes in catch efficiency. This case study provided a unique opportunity to demonstrate these effects over a nearly century-long time series, revealing that both small, incremental improvements as well as shifts in gear technology accumulate dramatic increases in catch efficiency over time. Raising awareness on the serious bias introduced by technological creep and associated risks is important considering the large number of stock assessments and fisheries policies that rely on CPUE data, especially in data-limited fisheries where fishery-independent data is not available.

### Depletion effect

Our modelling approach includes a novel local depletion effect that is relevant for other species where catch rates decline within a fishing season. Depletion effects may be common in fisheries where regulations, such as season or catch limits, lead to concentrated fishing effort at the opening of a fishery and subsequent fishing-out effects, especially for sedentary species. For lobster in Norway, the short fishing season (2–3 months) leads to intense fishing effort at the beginning of the season and decreasing catch rates during the season due to depletion of local lobster abundance^14^. Not including such information in CPUE standardization may therefore introduce bias, as shorter fishing seasons and increased focus on the season opening may create hyperstable catch rates despite a decline in abundance. Our proposed approach to model an explicit depletion effect in CPUE standardization may correct for the potential bias and improve CPUE estimation in a wide range of fisheries. Here, however, we also show that an alternate modelling approach that uses length of fishing season leads to a very similar result. This indicates that the results are robust and support the general conclusion; it also indicates that the relatively low-resolution data in our historic time series did not support the full estimation of depletion effects. Technological creep, thus, potentially remained underestimated. Recent CPUE data containing more detailed information on fishing activity throughout the season could help determine the degree of the depletion effect in Norway’s coastal lobster fishery.

### CPUE and population abundance

There are limitations when relating changes in CPUE index directly to changes in population abundance. Notably, CPUE reflects only the status of the lobster population that is currently harvested; it will not account for lobster in areas that are not being harvested. For example, a fishery may move or expand to deeper areas due to technical developments (e.g., vessel power) or because stock densities in inshore areas decrease. This is a concern in Norway’s coastal lobster fishery where fishing activity has expanded spatially in time into both deeper and more northern areas. For the period studied here, however, only anecdotal information is available. It was therefore not possible to link the depth effect on catchability observed in our experimental work to the CPUE time series. Our results, therefore, are a conservative estimate of technological creep, and data on spatial shifts in the fishery should be collected in future studies to strengthen the model.

### Technological creep

Data limitations and the resolution of the time series did not allow exploration of other potential causes of technological creep. Our study was, thus, focused on development of trap technology and did not include other factors that may drive technological creep^21–24^, such as increased engine power, Global Positioning System (GPS), echo-sounders, trap haulers, and easier access to information. The fishery has, in addition, changed from being dominated by commercial fishers to recreational fishers, although their catch rates are not statistically different^14^.

Introduction of two-chamber traps was the most important contribution to observed technological creep. Increased capture efficiency of these traps agrees with previous studies^18–20^. The development of an inner chamber in wooden traps in the 1980s ensured that more of the lobsters entering the trap were retained. Two-chamber traps made of steel frames and synthetic mesh did not increase CPUE significantly in our experiment. These traps, however, are more durable than wooden traps and, potentially, may increase fishing effort by reducing the need for maintenance. Our field experiment showed that two-chamber traps captured a higher proportion of large lobster than the older traps, matching the increased size of caught lobsters seen in historic data during the period when fishers began to use two-chambered traps. Larger sizes of harvested lobsters, thus, do not imply that the natural size-distribution in the lobster population has recovered. Rather, it is more likely that the larger funnel entrance made it possible for larger lobsters to enter the traps. The older traps were selecting for smaller high-priced (portion sized) lobster and effectively protected larger lobsters from being harvested. Although fishing pressure may have been high in the early part of the time series, large lobster with a high reproductive output^25,26^ were protected by the small funnel entrances. Protecting large, mature individuals in a stock can contribute to increased resilience and maintain productivity even under high fishing pressure^27,28^. The decrease in mean length following year 2000 indicates that the two-chambered traps have now largely depleted the larger size component. However, a maximum legal size limit (32 cm TL) introduced in 2017 for lobsters captured along the Norwegian Skagerrak coast is expected to reduce fishing pressure on large lobsters.

### The history of the lobster fishery

Despite the extraordinarily long time series available, the data does not cover the initial stages of the fishery. Already at the beginning of our time series in 1928, Norway’s coastal lobster fishery was well-developed with production for international export dating back to the 1600s^12^. In the beginning of 1800s, a common method of lobster capture was by long-handled pinchers in shallow water^12^, a method that would likely yield close to zero catch today. From 1876 to 1886, official landing statistics show yearly landings of 1–1.2 million lobsters a year. In 1928, 1.3 million (515 tons) lobsters were officially landed^12,29^. Today’s commercial lobster fishery is no longer economically viable with official landings of 41 tons in 2019. In contrast, catches of recreational fishers have increased over time, as recreational fishers are less restricted by economic constraints and can continue investing in improved fishing gear even though catch rates are low^30^. Recreational fishers are currently dominating the fishery with around 2/3 of the effort and catches in 2008^14^. Our case study exemplifies the challenges when studying coastal fisheries that often have a very long history of exploitation, making it especially difficult to trace back the development to a pristine state of the stock and resulting in often multi-faceted changes of the fishery over time.

### Concluding remarks

The standardized CPUE index corrected for technical creep highlights that the lobster population in Norway has suffered stronger decline due to prolonged over-harvesting than previous analyses have suggested. CPUE has declined by 92% over the last 90 years, indicating a dramatic decrease in the lobster population that has continued in recent decades. The severity of the decline becomes evident by virtue of a much longer data series than what is available for most fish stocks. Our study therefore underscores the importance of using a historic reference when studying changes in fish stocks and ecosystems over time, or else the magnitude of change may be masked by a shifting baseline in catchability^31,32^. The positive effects of experimental reserves on lobster density^33,34^ confirm the overfished state of Norway’s coastal lobster population, but also highlight the potential to rebuild if both commercial and recreational fisheries are adequately regulated.

In conclusion, our study demonstrates the importance of accounting for technological creep when estimating indices of catch efficiency and abundance. Using a time series that gives a unique historic perspective on a valuable lobster fishery, we demonstrated how development in trap design can affect the catch efficiency and thereby heavily bias fishery-dependent CPUE time series. Our conclusions underline the importance of careful standardization of fishery-dependent data to inform stock assessments and fisheries management.

## Methods

### Background on the lobster fishery in Norway

For over a century, the legal fishery for lobsters in Norway has mainly been conducted using traps. Until 2002, some fishing has taken place in the spring, but most catches were restricted to the autumn fishery starting on September 15th (until 1967) or October 1st (from 1968), and generally ending December 31st (November 30th since 2008 south of 62° N). Catch and effort data used in this study are from the autumn season only. Minimum landing size was introduced in 1879 (21 cm TL) and has increased over time: to 22 cm in 1964, 24 cm in 1992 and 25 cm in 2008 (for the Skagerrak coast with minor regional differences). The commercial lobster fishery was important for a long time, but the recreational fishery has been dominating in recent years^14,35^. There is a general lack of effort data, since there are no quota restrictions, and all commercial fishing vessels registered in Norway can legally fish for lobster. Moreover, a substantial proportion of the landings are not reported^14^. In 2017, an obligatory license system, for both commercial and recreational fishers, was introduced.

### Description of the data

#### CPUE time series

Since 1928, Norway’s Institute of Marine Research (IMR) has received standardized annual reports (between 22 and 94 reports per year with an average of 40) from volunteer commercial lobster fishers. The fishers report fishing period (start and stop date); fishing area; number of traps used; and total landings of legal lobster. The resolution of the dataset is the fishing season and information on catch rates per haul, detailed locations, depth or type of traps used were not available.

#### Desktop study/questionnaire survey

To identify the different lobster traps used over the last century in the Norwegian lobster fishery, a desktop study was conducted. A questionnaire survey was sent out to all active lobster fishers in the time series data base. Seven different trap types including pictures (identified through internet search and museums), with the option to nominate other trap types. The fishers were asked to identify the last two trap types they used and define the corresponding time periods of their use. They were also asked the time periods when each of the different trap types presented in the questionnaire was used in their fishing community. These data were used to generate a timeline of the most commonly used trap types since 1928.

#### Experimental fishing

Original traps were acquired for the four most common types. The traps were measured (size and funnel eye entrance) and, if needed, restored to their original state. The size of funnel web entrances (mean of length and height) for the different trap types were different: cylindrical traps 8.5 cm (n = 15, SE 0.1); wooden traps 10.1 cm (n = 9, SE 0.6); wooden two-chamber traps 13.3 cm (n = 10, SE 0.36); synthetic two-chamber traps; 12.0 cm (n = 17, SE 0.0, see Supplementary Table S4, Fig. S5 for additional trap details).

Field experiment was conducted in a typical lobster habitat in coastal Skagerrak (lat: 58.41, lon: 8.75) to estimate the catch efficiency of the four trap types. Fishing was conducted both inside and outside a lobster reserve to contrast different levels of lobster density^34^ with all four trap types.

Each trap was independently deployed based on a randomized design within pre-defined areas (more than 8° slope and between 8 and 30 m depth). Trap types were chronologically mixed at first deployment. A systematic hauling and deployment route was made for each hauling day, independent of trap type resulting in a random mix of trap type deployment. When a trap was hauled, it was moved to the next new randomly chosen waypoint for deployment. The survey lasted for 12 days in September 2014. All traps had a soak time of one night (~ 24 h). All catch was registered, and lobster length was recorded. Each Individual trap was inspected daily and repaired on site if necessary. If a retrieved trap was damaged and/or considered to have reduced catch efficiency, the data was not included in the experiment.

#### Other ancillary data

Since 1921, IMR has collected phenotypic data (including total length, weight, sex) on lobster. Initially, all such data was collected in the coastal areas outside the IMR Flødevigen research station in Arendal (~ 100 lobsters sampled per year), until in 1938 the counties of Agder and Telemark were included. In 1962, inner Skagerrak (Hvaler archipelago) was added as an area of data collection. In 2004, the fishers were trained to measure and report the first 300 lobsters captured each season. From 1949 to 2019, the mean number of lobsters measured annually was 2200. All lobsters captured were registered, including individuals below legal size. When obligatory escape vents were introduced in 2008, the fishers received an exemption from the regulation to continue the time series. In total, the time series contains ~ 150,000 lobster observations.

### Statistical analysis

We performed a CPUE standardization to derive a standardized index of annual CPUE representing changes in lobster abundance. However, the core data—the CPUE time series—only contained information on the number of lobsters caught during each fisher’s fishing season (date start and stop) with no information on technical parameters affecting fishing efficiency; notably, the trap type and lobster catching efficiency. CPUE standardization was, therefore, conducted in several steps:

#### Estimating trap type usage over time

Missing information on trap type was derived from the questionnaire survey data. However, the questionnaire would only provide coarse, annual level trap type use, i.e. the average proportion of trap type used in a year, not the individual level information needed in the CPUE analysis. Therefore, the number of trap types used in observation *i* ($${Number}_{i, trap})$$ was initially obtained by taking the product of each fisher’s trap count ($${Trap\_count}_{i})$$ and the proportion of each trap type in the corresponding year ($${p}_{{year}_{i}}$$).

1 $$\begin{array}{c}{Number}_{i, trap}= {Tra{p\_count}_{i}p}_{{year}_{i}},\end{array}$$

#### Estimating trap type catchability

While the above step allows imputing the trap types used by each fisher, the catch efficiency of each trap type is unknown. Catchability of trap types was therefore estimated by fitting a generalized linear mixed effect model to lobster catch data obtained from the field experiment:

2 $${N}_{i}\sim {T}_{i}+s\left({D}_{i}\right)+{(1|A}_{i}),$$

where ${N}_{i}$ is the number of lobsters caught in observation $i$ with trap type $T$ at bottom depth $D$ in area type $A$. A smoothing spline was used on $D$ to account for expected nonlinear relationship between catch rate and depth. The effect of area type *A* was modelled as random effect acting on the intercept term (1|A) in order to acknowledge differences in catchability between the reserve and control sites, and for the possibility to predicting lobster catch at other random sites. The model was fitted in glmmTMB^36^ with a Poisson distribution (log-link), and residual distribution and model dispersion were evaluated to validate the fit (Supplementary Fig. S6).

Catchability of each trap type was then derived by calculating its marginal effect, i.e. predicting the number of lobsters caught by each trap type at an average depth level and for a random area (i.e. the area effect is put at 0).

#### Deriving a standardized unit of effort

Combining information from steps 1 and 2, we calculated a standardized unit of effort (${Std\_effort}_{i}$) for each observation *i* to properly account for the changes in trap type use, fishing efficiency, and fishing season length.

3 $$\begin{array}{c}St{d\_effort}_{i} ={Fishing\_days}_{i}\times \sum_{trap}{catchability}_{i,trap}\times {Number}_{i, trap},\end{array}$$

with ${Fishing\_days}_{i}$ as the number of fishing days per season and fisher, and $\sum_{trap}{catchability}_{i,trap}\times {Number}_{i, trap}$ the sum of catchability per trap type used and total number of traps. This was contrasted with the unstandardized unit of effort (${raw\_effort}_{i})$ which only accounted for the effect of fishing duration $({Fishing\_days}_{i})$ and trap count (${Trap\_count}_{i}$) but not the effect of trap type efficiency:

4 $$\begin{array}{c}{Raw\_effort}_{i} ={Fishing\_days}_{i}\times Trap\_count_{i}.\end{array}$$

#### Deriving the corrected CPUE

The corrected CPUE for each observation *i* (i.e. lobster catch per day and per trap that was adjusted for technological creep) was then calculated as:

5 $$\begin{array}{c}{CPUE}_{i,corrected}=\frac{N{b\_lobster}_{i} }{St{d\_effort}_{i}},\end{array}$$

where ${Nb\_lobster}_{i}$ is the total number of lobster caught for observation *i.*

This is to be contrasted with an uncorrected CPUE for observation *i*, where CPUE was not adjusted for technological creep:

6 $$\begin{array}{c}{CPUE}_{i, uncorrected}= \frac{N{b\_lobster}_{i}}{{raw\_effort}_{i}}.\end{array}$$

#### Standardizing the corrected CPUE data and calculating an index of abundance

The above corrected CPUE_i_ corresponds to the average CPUE per day and trap based on the duration of the fishing season for observation *i.* However, the analysis of logbooks (n = 501 fishers, covering 106,364 observations from 2008 to 2019) of daily catches from lobster fishers^14^ by fishing area indicated that the daily lobster catch rate decreased over the season (Supplementary Figs. S7, S8 for area definition). Therefore, in the following step, we tried to further standardize the corrected CPUE to account for the seasonal depletion of lobster catch rate, as well as the effects of some available environmental covariates.

Assuming the seasonal depletion of daily lobster catch rate per trap, *cr*_*t*_, follows an exponential decreasing function:

7 $$\begin{array}{c}{cr}_{t}=\alpha {e}^{-\beta t},\end{array}$$

where *α* is the intercept (i.e., the catch rate at the beginning of the fishing season) and *β* the slope of the declining catch rate over the course of the season, *t.* ${CPUE}_{i,corrected}$ from observation *i* from the interview time series can then be modelled as the average catch rate over the season, from the start date, *start*_*i*_, until the last fishing date *end*_*i*_ (see Supplementary Eq. (S1) for the detailed derivation of this equation) using the equation below:

8 $$\begin{array}{c}{CPUE}_{i,corrected}\sim \frac{\alpha }{{\beta (end}_{i}-{start}_{i})}\left({e}^{-\beta {start}_{i}}-{e}^{-\beta {end}_{i}}\right).\end{array}$$

By replacing ${\alpha }^{^{\prime}}=\frac{\alpha }{\beta }$, the above equation can be rewritten as

9 $$\begin{array}{c}{CPUE}_{i,corrected}\sim \frac{{\alpha }^{^{\prime}}}{{(end}_{i}-{start}_{i})}\left({e}^{-\beta {start}_{i}}-{e}^{-\beta {end}_{i}}\right).\end{array}$$

We then modelled ${\alpha }^{^{\prime}}$ and $\beta$ as functions of covariates. Year indicates the year effect and is modelled as a categorical variable. *(1|Area)* indicates that the *Area* effect is modelled as a normally distributed random intercept:

10 $$\begin{array}{*{20}c} {\begin{array}{*{20}c} {\alpha^{\prime}\sim Year + \left( {1{|}Area} \right)} \\ \end{array} } \\ {\beta \sim 1} \\ \end{array} .$$

The model was fitted in TMB^37^ with a log-link gamma distribution. Once the model was run and passed the residual diagnostics test (Supplementary Fig. S2), it was then used to calculate an index of abundance. The derived index was named “Standardized corrected CPUE” and was calculated based on the predicted $\widehat{CPUE}$ at the beginning of the fishing season, for each year, on population level (i.e. value of $Area$ fixed to 0).

#### Accounting for uncertainty in step 1 and 2 in the index of abundance calculation

To incorporate the uncertainty associated with the estimated trap type use over time and trap type catchability, we resampled values of ${Number}_{i, trap}$ and ${catchability}_{i,trap}$ 1000 times. ${Number}_{i, trap}$ was sampled from a multinomial distribution with sample size equal to the individual’s trap count (${Trap\_count}_{i})$ and probability equal to the proportion of each trap type in for the corresponding year (${p}_{{year}_{i}}$).

11 $$\begin{array}{c}{Number}_{i, trap}\sim Multinomial\left({Trap\_count}_{i},{p}_{{year}_{i}}\right) .\end{array}$$

${catchability}_{i,trap}$ was resampled from a normal distribution centred around the estimated mean and standard deviation. For each of the 1000 resamples values, steps 1–5 were repeated to produce 1000 indices of lobster abundance (Table S3).

#### Alternate CPUE standardization models and abundance indices

To examine the impacts of technological creep (step 1–4), and other CPUE standardization step (the seasonal depletion effect described in step 5) on the abundance indices, we applied alternative models which either included or did not include technological creep to derive lobster abundance indices. The terms “corrected”/“uncorrected” were already introduced above to indicate the presence/absence of correction related to technological creep. Similarly, we used the terms “standardized”/“unstandardized” for the presence/absence of the other CPUE standardization process. For “unstandardized” models, indices of abundance were simply obtained by calculating the mean “corrected”/“uncorrected” CPUE by year.

Lobster catch was also analysed with a more traditional approach that did not explicitly account for depletion effects but used fishing duration (${Fishing\_days}_{i})$ as a variable in addition to the Year and Area effects, and included the catchability correction ($\sum_{trap}{catchability}_{i,trap}\times {Number}_{i, trap})$ as offset:

12 $${Nb_{{lobster_{i} }} = Year + \left( {1|Area} \right) + s\left( {Fishing_{{days_{i} }} } \right) + offset\left( {\log \left( {\sum\limits_{{trap}} {catchability_{{i,trap}} } \times Number_{{i,trap}} } \right)} \right).}$$

The model was implemented in glmmTMB with a log-link gamma distribution with Year effect modelled as factor, Area effect as random effect acting on the intercept, and with a smoothing spline structure on the effect of total fishing days (Supplementary Fig. S6). The abundance index was derived based on predicted lobster abundance at the start of the fishing season each year, at population level ($Area$ effect set at 0). The derived index was named “Standardized corrected CPUE (alt)” (Supplementary Fig. S1).

#### Making all abundance indices comparable

All indices were subsequently divided by the predicted $CPUE$ at the beginning of the time series (1928) or during a period where fishers switch from one-chamber to two-chamber trap types (1980) to show the depletion rate compared to these two periods.

## Supplementary Information

Supplementary Information.

## Data Availability

Data supporting the findings of this study are available from the corresponding author upon request.
