# Supplementary material for: Technological creep masks continued decline in a lobster (Homarus gammarus) fishery over a century
Source: Sci Rep. 2022 Feb 28;12:3318. doi: 10.1038/s41598-022-07293-2 (PMC8885706; doi:10.1038/s41598-022-07293-2)
Supplement: Supplementary file 1 — Supplementary Information. [file 41598_2022_7293_MOESM1_ESM.pdf]

Supplementary information for the manuscript:

Technological creep masks continued decline in a lobster (*Homarus gammarus*) fishery over a century

Alf Ring Kleiven[1], Sigurd Heiberg Espeland[1,2], Stian Stiansen[1], Kotaro Ono [1], Fabian Zimmermann[1], Esben Moland Olsen[1,2]

1 Norwegian Institute of Marine Research, Bergen, Norway

2 Centre for Coastal Research, Department of Natural Sciences, University of Agder, Kristiansand, Norway

Content:

1) Table S1. Table of parameters estimated from a GLMM on experiment fishing data

2) Table S2. Estimated lobster population depletion rate (in %) compared to 1980 and 1928 for the five CPUE standardization models explored in this study

3) Table S3. Estimated time series of abundance index for the five CPUE standardization models.

4) Table S4. Technical data for traps used in the study.

5) Figure S1. Lobster abundance indices from the depletion model compared to the alternate model based on GLMM

6) Figure S2. Residual diagnostics for the lobster depletion model.

7) Figure S3. Residual diagnostics for the alternative lobster depletion model fitted in glmmTMB.

8) Figure S4. Estimated Year and season length effect from the alternative lobster depletion model fitted in glmmTMB.

9) Figure S5. Traps identified as most common throughout the time series and used in the experimental test fishing.

10) Figure S6. Residual diagnostics for the glmmTMB model to estimate trap type catchability.

11) Figure S7. Lobster CPUE by area and year from the logbook data

12) Figure S8. Map of the sample areas for the logbook data.

13) Equation S1. Mathematical derivation of the depletion model

Table S1: Table of parameters estimated from a GLMM on experiment fishing data and corresponding standard errors and p-values indicating significance.

Variable name	Estimate	Std. Error	Pr(>|z|)	Significance
Intercept	-2.300	0.542	0.000	***
Wooden trap	0.268	0.336	0.426	
wooden two-chamber trap	1.238	0.284	0.000	***
synthetic two-chamber trap	1.397	0.283	0.000	***
ns(depth, df = 3)1	0.693	0.370	0.061	.
ns(depth, df = 3)2	1.161	1.127	0.303	
ns(depth, df = 3)3	-0.006	0.673	0.993	
Variance(area)	0.01929	--	--	

*** indicates significance at alpha of 0.001,
. indicates significance at alpha of 0.1

Table S2. Estimated lobster population depletion rate (in %) compared to 1980 and 1928 for the five CPUE standardization models explored in this study:

Model	depletion1980	depletion1928
Unstandardized uncorrected CPUE	82.98	19.04
Unstandardized corrected CPUE	32.16	5.02
Standardized uncorrected CPUE	107.89	29.94
Standardized corrected CPUE	42.56	7.64
Standardized corrected CPUE (alt)	45.91	7.68

Table S3. Estimate time series of abundance index for the five models (Unstandardized uncorrected CPUE, Unstandardized corrected CPUE, Standardized uncorrected CPUE, Standardized corrected CPUE, and Standardized corrected CPUE (alt)) along with the 95% confidence interval (low_scl and upp_scl). Confidence interval around the Standardized corrected CPUE accounts for the uncertainty in traps types and number based on the resampling procedure described in the main text.

model	Year	Index	low_scl	upp_scl
Standardized corrected CPUE	1928	1.00	0.66	1.34
Standardized corrected CPUE	1929	0.94	0.52	1.36
Standardized corrected CPUE	1930	0.76	0.60	0.93
Standardized corrected CPUE	1931	0.86	0.70	1.02
Standardized corrected CPUE	1932	0.85	0.68	1.03
Standardized corrected CPUE	1933	0.69	0.57	0.82
Standardized corrected CPUE	1934	0.55	0.44	0.66
Standardized corrected CPUE	1935	0.53	0.39	0.66
Standardized corrected CPUE	1936	0.72	0.57	0.87
Standardized corrected CPUE	1937	0.75	0.02	1.49
Standardized corrected CPUE	1938	0.46	0.35	0.57
Standardized corrected CPUE	1939	0.85	0.66	1.03
Standardized corrected CPUE	1940	0.78	0.59	0.98
Standardized corrected CPUE	1941	0.64	0.51	0.76
Standardized corrected CPUE	1942	0.93	0.74	1.12
Standardized corrected CPUE	1943	0.73	0.60	0.86
Standardized corrected CPUE	1944	0.90	0.63	1.17

Standardized corrected CPUE	1945	1.39	1.11	1.67
Standardized corrected CPUE	1946	0.93	0.76	1.10
Standardized corrected CPUE	1947	0.80	0.65	0.94
Standardized corrected CPUE	1948	0.78	0.67	0.90
Standardized corrected CPUE	1949	1.02	0.86	1.17
Standardized corrected CPUE	1950	0.75	0.64	0.87
Standardized corrected CPUE	1951	0.77	0.64	0.89
Standardized corrected CPUE	1952	0.81	0.67	0.94
Standardized corrected CPUE	1953	0.70	0.56	0.83
Standardized corrected CPUE	1954	0.77	0.61	0.92
Standardized corrected CPUE	1955	0.55	0.46	0.65
Standardized corrected CPUE	1956	0.98	0.76	1.20
Standardized corrected CPUE	1957	0.45	0.36	0.54
Standardized corrected CPUE	1958	0.59	0.50	0.68
Standardized corrected CPUE	1959	0.50	0.42	0.57
Standardized corrected CPUE	1960	0.55	0.47	0.63
Standardized corrected CPUE	1961	0.47	0.39	0.56
Standardized corrected CPUE	1962	0.52	0.44	0.60
Standardized corrected CPUE	1963	0.49	0.41	0.57
Standardized corrected CPUE	1964	0.43	0.35	0.52
Standardized corrected CPUE	1965	0.30	0.26	0.35
Standardized corrected CPUE	1966	0.37	0.31	0.42
Standardized corrected CPUE	1967	0.28	0.22	0.34
Standardized corrected CPUE	1968	0.26	0.22	0.31
Standardized corrected CPUE	1969	0.35	0.30	0.40
Standardized corrected CPUE	1970	0.36	0.29	0.43
Standardized corrected CPUE	1971	0.19	0.16	0.22
Standardized corrected CPUE	1972	0.26	0.22	0.31
Standardized corrected CPUE	1973	0.27	0.23	0.31
Standardized corrected CPUE	1974	0.24	0.19	0.28
Standardized corrected CPUE	1975	0.27	0.24	0.29
Standardized corrected CPUE	1976	0.30	0.28	0.31
Standardized corrected CPUE	1977	0.31	0.29	0.33
Standardized corrected CPUE	1978	0.24	0.22	0.26
Standardized corrected CPUE	1979	0.23	0.21	0.25
Standardized corrected CPUE	1980	0.18	0.17	0.19
Standardized corrected CPUE	1981	0.20	0.18	0.22
Standardized corrected CPUE	1982	0.18	0.17	0.20
Standardized corrected CPUE	1983	0.19	0.17	0.20
Standardized corrected CPUE	1984	0.18	0.16	0.19
Standardized corrected CPUE	1985	0.15	0.14	0.16
Standardized corrected CPUE	1986	0.15	0.14	0.17
Standardized corrected CPUE	1987	0.15	0.14	0.16
Standardized corrected CPUE	1988	0.14	0.13	0.15
Standardized corrected CPUE	1989	0.17	0.16	0.18
Standardized corrected CPUE	1990	0.12	0.11	0.13
Standardized corrected CPUE	1991	0.13	0.12	0.14
Standardized corrected CPUE	1992	0.09	0.09	0.10

Standardized corrected CPUE	1993	0.11	0.10	0.12
Standardized corrected CPUE	1994	0.13	0.12	0.14
Standardized corrected CPUE	1995	0.15	0.14	0.16
Standardized corrected CPUE	1996	0.13	0.12	0.14
Standardized corrected CPUE	1997	0.13	0.13	0.14
Standardized corrected CPUE	1998	0.13	0.12	0.14
Standardized corrected CPUE	1999	0.12	0.11	0.13
Standardized corrected CPUE	2000	0.07	0.07	0.07
Standardized corrected CPUE	2001	0.07	0.07	0.08
Standardized corrected CPUE	2002	0.08	0.08	0.09
Standardized corrected CPUE	2003	0.11	0.10	0.12
Standardized corrected CPUE	2004	0.09	0.09	0.10
Standardized corrected CPUE	2005	0.10	0.09	0.11
Standardized corrected CPUE	2006	0.10	0.09	0.11
Standardized corrected CPUE	2007	0.09	0.09	0.10
Standardized corrected CPUE	2008	0.08	0.08	0.09
Standardized corrected CPUE	2009	0.13	0.12	0.14
Standardized corrected CPUE	2010	0.10	0.09	0.11
Standardized corrected CPUE	2011	0.10	0.10	0.11
Standardized corrected CPUE	2012	0.09	0.08	0.10
Standardized corrected CPUE	2013	0.09	0.09	0.10
Standardized corrected CPUE	2014	0.07	0.07	0.08
Standardized corrected CPUE	2015	0.08	0.07	0.09
Standardized corrected CPUE	2016	0.07	0.06	0.08
Standardized corrected CPUE	2017	0.08	0.07	0.09
Standardized corrected CPUE	2018	0.08	0.07	0.09
Standardized corrected CPUE	2019	0.08	0.07	0.09
Standardized uncorrected CPUE	1928	1.00	0.69	1.31
Standardized uncorrected CPUE	1929	0.94	0.66	1.23
Standardized uncorrected CPUE	1930	0.79	0.57	1.01
Standardized uncorrected CPUE	1931	0.89	0.64	1.13
Standardized uncorrected CPUE	1932	0.88	0.65	1.11
Standardized uncorrected CPUE	1933	0.72	0.54	0.90
Standardized uncorrected CPUE	1934	0.57	0.40	0.73
Standardized uncorrected CPUE	1935	0.54	0.38	0.70
Standardized uncorrected CPUE	1936	0.74	0.53	0.95
Standardized uncorrected CPUE	1937	0.76	0.56	0.97
Standardized uncorrected CPUE	1938	0.48	0.34	0.61
Standardized uncorrected CPUE	1939	0.87	0.63	1.11
Standardized uncorrected CPUE	1940	0.80	0.58	1.03
Standardized uncorrected CPUE	1941	0.66	0.48	0.84
Standardized uncorrected CPUE	1942	0.96	0.68	1.24
Standardized uncorrected CPUE	1943	0.76	0.56	0.96
Standardized uncorrected CPUE	1944	0.93	0.66	1.20
Standardized uncorrected CPUE	1945	1.44	1.05	1.83
Standardized uncorrected CPUE	1946	0.98	0.72	1.24
Standardized uncorrected CPUE	1947	0.83	0.65	1.02
Standardized uncorrected CPUE	1948	0.84	0.65	1.03

Standardized uncorrected CPUE	1949	1.08	0.82	1.35
Standardized uncorrected CPUE	1950	0.84	0.62	1.07
Standardized uncorrected CPUE	1951	0.86	0.63	1.09
Standardized uncorrected CPUE	1952	0.91	0.64	1.17
Standardized uncorrected CPUE	1953	0.78	0.53	1.04
Standardized uncorrected CPUE	1954	0.85	0.58	1.13
Standardized uncorrected CPUE	1955	0.62	0.45	0.79
Standardized uncorrected CPUE	1956	1.10	0.72	1.47
Standardized uncorrected CPUE	1957	0.51	0.33	0.68
Standardized uncorrected CPUE	1958	0.67	0.49	0.84
Standardized uncorrected CPUE	1959	0.55	0.42	0.69
Standardized uncorrected CPUE	1960	0.64	0.50	0.79
Standardized uncorrected CPUE	1961	0.55	0.41	0.69
Standardized uncorrected CPUE	1962	0.61	0.46	0.75
Standardized uncorrected CPUE	1963	0.57	0.42	0.71
Standardized uncorrected CPUE	1964	0.50	0.37	0.63
Standardized uncorrected CPUE	1965	0.40	0.29	0.51
Standardized uncorrected CPUE	1966	0.49	0.35	0.62
Standardized uncorrected CPUE	1967	0.37	0.25	0.49
Standardized uncorrected CPUE	1968	0.35	0.24	0.46
Standardized uncorrected CPUE	1969	0.47	0.34	0.59
Standardized uncorrected CPUE	1970	0.48	0.33	0.64
Standardized uncorrected CPUE	1971	0.26	0.18	0.33
Standardized uncorrected CPUE	1972	0.36	0.25	0.46
Standardized uncorrected CPUE	1973	0.37	0.26	0.47
Standardized uncorrected CPUE	1974	0.32	0.22	0.43
Standardized uncorrected CPUE	1975	0.36	0.29	0.43
Standardized uncorrected CPUE	1976	0.40	0.36	0.45
Standardized uncorrected CPUE	1977	0.42	0.37	0.47
Standardized uncorrected CPUE	1978	0.33	0.28	0.37
Standardized uncorrected CPUE	1979	0.31	0.27	0.35
Standardized uncorrected CPUE	1980	0.28	0.24	0.32
Standardized uncorrected CPUE	1981	0.31	0.26	0.35
Standardized uncorrected CPUE	1982	0.32	0.27	0.36
Standardized uncorrected CPUE	1983	0.32	0.28	0.36
Standardized uncorrected CPUE	1984	0.31	0.26	0.35
Standardized uncorrected CPUE	1985	0.29	0.25	0.33
Standardized uncorrected CPUE	1986	0.30	0.25	0.34
Standardized uncorrected CPUE	1987	0.29	0.25	0.33
Standardized uncorrected CPUE	1988	0.27	0.23	0.31
Standardized uncorrected CPUE	1989	0.32	0.27	0.37
Standardized uncorrected CPUE	1990	0.31	0.26	0.36
Standardized uncorrected CPUE	1991	0.34	0.29	0.39
Standardized uncorrected CPUE	1992	0.25	0.21	0.29
Standardized uncorrected CPUE	1993	0.29	0.24	0.33
Standardized uncorrected CPUE	1994	0.35	0.29	0.40
Standardized uncorrected CPUE	1995	0.39	0.33	0.45
Standardized uncorrected CPUE	1996	0.33	0.28	0.38

Standardized uncorrected CPUE	1997	0.35	0.30	0.40
Standardized uncorrected CPUE	1998	0.33	0.29	0.38
Standardized uncorrected CPUE	1999	0.32	0.28	0.36
Standardized uncorrected CPUE	2000	0.23	0.20	0.26
Standardized uncorrected CPUE	2001	0.24	0.20	0.27
Standardized uncorrected CPUE	2002	0.27	0.23	0.31
Standardized uncorrected CPUE	2003	0.37	0.32	0.42
Standardized uncorrected CPUE	2004	0.31	0.27	0.36
Standardized uncorrected CPUE	2005	0.35	0.30	0.41
Standardized uncorrected CPUE	2006	0.35	0.30	0.41
Standardized uncorrected CPUE	2007	0.33	0.28	0.37
Standardized uncorrected CPUE	2008	0.30	0.25	0.34
Standardized uncorrected CPUE	2009	0.46	0.40	0.53
Standardized uncorrected CPUE	2010	0.39	0.34	0.44
Standardized uncorrected CPUE	2011	0.40	0.35	0.46
Standardized uncorrected CPUE	2012	0.34	0.29	0.39
Standardized uncorrected CPUE	2013	0.36	0.31	0.42
Standardized uncorrected CPUE	2014	0.28	0.24	0.32
Standardized uncorrected CPUE	2015	0.31	0.26	0.35
Standardized uncorrected CPUE	2016	0.28	0.24	0.32
Standardized uncorrected CPUE	2017	0.31	0.26	0.37
Standardized uncorrected CPUE	2018	0.30	0.24	0.36
Standardized uncorrected CPUE	2019	0.30	0.24	0.36
Standardized corrected CPUE (alt)	1928	1.00	0.32	1.68
Standardized corrected CPUE (alt)	1929	0.93	0.30	1.56
Standardized corrected CPUE (alt)	1930	0.77	0.25	1.29
Standardized corrected CPUE (alt)	1931	0.86	0.28	1.44
Standardized corrected CPUE (alt)	1932	0.86	0.29	1.42
Standardized corrected CPUE (alt)	1933	0.69	0.23	1.15
Standardized corrected CPUE (alt)	1934	0.56	0.18	0.94
Standardized corrected CPUE (alt)	1935	0.53	0.17	0.90
Standardized corrected CPUE (alt)	1936	0.71	0.23	1.19
Standardized corrected CPUE (alt)	1937	0.74	0.25	1.23
Standardized corrected CPUE (alt)	1938	0.47	0.15	0.78
Standardized corrected CPUE (alt)	1939	0.85	0.28	1.42
Standardized corrected CPUE (alt)	1940	0.76	0.25	1.28
Standardized corrected CPUE (alt)	1941	0.64	0.21	1.07
Standardized corrected CPUE (alt)	1942	0.95	0.31	1.60
Standardized corrected CPUE (alt)	1943	0.75	0.25	1.25
Standardized corrected CPUE (alt)	1944	0.92	0.30	1.54
Standardized corrected CPUE (alt)	1945	1.40	0.47	2.34
Standardized corrected CPUE (alt)	1946	0.94	0.32	1.57
Standardized corrected CPUE (alt)	1947	0.80	0.28	1.32
Standardized corrected CPUE (alt)	1948	0.79	0.28	1.30
Standardized corrected CPUE (alt)	1949	1.02	0.35	1.69
Standardized corrected CPUE (alt)	1950	0.76	0.26	1.27
Standardized corrected CPUE (alt)	1951	0.77	0.26	1.29
Standardized corrected CPUE (alt)	1952	0.83	0.27	1.39

Standardized corrected CPUE (alt)	1953	0.72	0.22	1.22
Standardized corrected CPUE (alt)	1954	0.77	0.24	1.30
Standardized corrected CPUE (alt)	1955	0.49	0.16	0.82
Standardized corrected CPUE (alt)	1956	0.99	0.30	1.69
Standardized corrected CPUE (alt)	1957	0.48	0.14	0.81
Standardized corrected CPUE (alt)	1958	0.57	0.19	0.95
Standardized corrected CPUE (alt)	1959	0.50	0.17	0.83
Standardized corrected CPUE (alt)	1960	0.57	0.20	0.94
Standardized corrected CPUE (alt)	1961	0.47	0.16	0.78
Standardized corrected CPUE (alt)	1962	0.51	0.18	0.85
Standardized corrected CPUE (alt)	1963	0.49	0.17	0.82
Standardized corrected CPUE (alt)	1964	0.43	0.14	0.71
Standardized corrected CPUE (alt)	1965	0.28	0.09	0.47
Standardized corrected CPUE (alt)	1966	0.37	0.12	0.62
Standardized corrected CPUE (alt)	1967	0.27	0.08	0.45
Standardized corrected CPUE (alt)	1968	0.27	0.08	0.45
Standardized corrected CPUE (alt)	1969	0.37	0.12	0.62
Standardized corrected CPUE (alt)	1970	0.34	0.11	0.58
Standardized corrected CPUE (alt)	1971	0.20	0.06	0.33
Standardized corrected CPUE (alt)	1972	0.26	0.09	0.44
Standardized corrected CPUE (alt)	1973	0.28	0.09	0.46
Standardized corrected CPUE (alt)	1974	0.25	0.08	0.42
Standardized corrected CPUE (alt)	1975	0.26	0.09	0.43
Standardized corrected CPUE (alt)	1976	0.27	0.10	0.44
Standardized corrected CPUE (alt)	1977	0.30	0.11	0.48
Standardized corrected CPUE (alt)	1978	0.23	0.08	0.37
Standardized corrected CPUE (alt)	1979	0.23	0.08	0.37
Standardized corrected CPUE (alt)	1980	0.17	0.06	0.27
Standardized corrected CPUE (alt)	1981	0.20	0.07	0.33
Standardized corrected CPUE (alt)	1982	0.18	0.07	0.30
Standardized corrected CPUE (alt)	1983	0.18	0.07	0.30
Standardized corrected CPUE (alt)	1984	0.17	0.06	0.28
Standardized corrected CPUE (alt)	1985	0.15	0.06	0.25
Standardized corrected CPUE (alt)	1986	0.15	0.06	0.25
Standardized corrected CPUE (alt)	1987	0.14	0.05	0.22
Standardized corrected CPUE (alt)	1988	0.13	0.05	0.21
Standardized corrected CPUE (alt)	1989	0.17	0.06	0.27
Standardized corrected CPUE (alt)	1990	0.12	0.04	0.20
Standardized corrected CPUE (alt)	1991	0.12	0.05	0.20
Standardized corrected CPUE (alt)	1992	0.10	0.04	0.16
Standardized corrected CPUE (alt)	1993	0.11	0.04	0.18
Standardized corrected CPUE (alt)	1994	0.13	0.05	0.21
Standardized corrected CPUE (alt)	1995	0.15	0.05	0.24
Standardized corrected CPUE (alt)	1996	0.13	0.05	0.21
Standardized corrected CPUE (alt)	1997	0.14	0.05	0.22
Standardized corrected CPUE (alt)	1998	0.13	0.05	0.22
Standardized corrected CPUE (alt)	1999	0.12	0.05	0.20
Standardized corrected CPUE (alt)	2000	0.07	0.03	0.12

Standardized corrected CPUE (alt)	2001	0.07	0.03	0.12
Standardized corrected CPUE (alt)	2002	0.08	0.03	0.13
Standardized corrected CPUE (alt)	2003	0.11	0.04	0.18
Standardized corrected CPUE (alt)	2004	0.10	0.04	0.16
Standardized corrected CPUE (alt)	2005	0.10	0.04	0.16
Standardized corrected CPUE (alt)	2006	0.09	0.03	0.15
Standardized corrected CPUE (alt)	2007	0.09	0.03	0.15
Standardized corrected CPUE (alt)	2008	0.08	0.03	0.13
Standardized corrected CPUE (alt)	2009	0.10	0.04	0.16
Standardized corrected CPUE (alt)	2010	0.10	0.04	0.17
Standardized corrected CPUE (alt)	2011	0.11	0.04	0.17
Standardized corrected CPUE (alt)	2012	0.09	0.03	0.15
Standardized corrected CPUE (alt)	2013	0.10	0.04	0.16
Standardized corrected CPUE (alt)	2014	0.08	0.03	0.12
Standardized corrected CPUE (alt)	2015	0.08	0.03	0.13
Standardized corrected CPUE (alt)	2016	0.07	0.03	0.12
Standardized corrected CPUE (alt)	2017	0.08	0.03	0.13
Standardized corrected CPUE (alt)	2018	0.08	0.03	0.13
Standardized corrected CPUE (alt)	2019	0.08	0.03	0.13
Unstandardized uncorrected CPUE	1928	1.00	-0.25	2.25
Unstandardized uncorrected CPUE	1929	0.90	0.16	1.64
Unstandardized uncorrected CPUE	1930	0.58	0.13	1.03
Unstandardized uncorrected CPUE	1931	0.71	0.18	1.25
Unstandardized uncorrected CPUE	1932	0.66	0.18	1.13
Unstandardized uncorrected CPUE	1933	0.57	0.08	1.05
Unstandardized uncorrected CPUE	1934	0.44	0.05	0.83
Unstandardized uncorrected CPUE	1935	0.48	-0.02	0.99
Unstandardized uncorrected CPUE	1936	0.66	0.06	1.26
Unstandardized uncorrected CPUE	1937	0.69	0.10	1.28
Unstandardized uncorrected CPUE	1938	0.41	0.09	0.74
Unstandardized uncorrected CPUE	1939	0.86	-0.17	1.88
Unstandardized uncorrected CPUE	1940	0.65	0.09	1.22
Unstandardized uncorrected CPUE	1941	0.58	-0.07	1.23
Unstandardized uncorrected CPUE	1942	0.72	0.17	1.26
Unstandardized uncorrected CPUE	1943	0.57	0.15	0.99
Unstandardized uncorrected CPUE	1944	0.72	0.19	1.24
Unstandardized uncorrected CPUE	1945	0.98	0.24	1.71
Unstandardized uncorrected CPUE	1946	0.79	0.01	1.56
Unstandardized uncorrected CPUE	1947	0.67	0.10	1.24
Unstandardized uncorrected CPUE	1948	0.66	0.16	1.15
Unstandardized uncorrected CPUE	1949	0.85	0.22	1.48
Unstandardized uncorrected CPUE	1950	0.64	0.17	1.10
Unstandardized uncorrected CPUE	1951	0.63	0.19	1.06
Unstandardized uncorrected CPUE	1952	0.69	0.10	1.29
Unstandardized uncorrected CPUE	1953	0.65	0.08	1.23
Unstandardized uncorrected CPUE	1954	0.67	0.12	1.23
Unstandardized uncorrected CPUE	1955	0.51	-0.14	1.16
Unstandardized uncorrected CPUE	1956	0.94	-0.14	2.01

Unstandardized uncorrected CPUE	1957	0.40	-0.07	0.87
Unstandardized uncorrected CPUE	1958	0.48	0.05	0.90
Unstandardized uncorrected CPUE	1959	0.39	-0.06	0.83
Unstandardized uncorrected CPUE	1960	0.52	0.12	0.93
Unstandardized uncorrected CPUE	1961	0.40	0.00	0.81
Unstandardized uncorrected CPUE	1962	0.49	-0.13	1.12
Unstandardized uncorrected CPUE	1963	0.43	0.03	0.83
Unstandardized uncorrected CPUE	1964	0.44	-0.18	1.07
Unstandardized uncorrected CPUE	1965	0.33	-0.19	0.85
Unstandardized uncorrected CPUE	1966	0.44	0.01	0.86
Unstandardized uncorrected CPUE	1967	0.38	-0.41	1.17
Unstandardized uncorrected CPUE	1968	0.27	-0.05	0.58
Unstandardized uncorrected CPUE	1969	0.36	-0.01	0.73
Unstandardized uncorrected CPUE	1970	0.39	0.07	0.71
Unstandardized uncorrected CPUE	1971	0.21	0.02	0.40
Unstandardized uncorrected CPUE	1972	0.29	0.08	0.50
Unstandardized uncorrected CPUE	1973	0.34	-0.24	0.92
Unstandardized uncorrected CPUE	1974	0.28	-0.01	0.56
Unstandardized uncorrected CPUE	1975	0.31	0.02	0.59
Unstandardized uncorrected CPUE	1976	0.33	-0.14	0.81
Unstandardized uncorrected CPUE	1977	0.35	-0.11	0.81
Unstandardized uncorrected CPUE	1978	0.28	0.02	0.54
Unstandardized uncorrected CPUE	1979	0.27	0.00	0.54
Unstandardized uncorrected CPUE	1980	0.23	-0.07	0.53
Unstandardized uncorrected CPUE	1981	0.25	-0.06	0.55
Unstandardized uncorrected CPUE	1982	0.26	-0.17	0.70
Unstandardized uncorrected CPUE	1983	0.24	-0.10	0.58
Unstandardized uncorrected CPUE	1984	0.25	-0.15	0.66
Unstandardized uncorrected CPUE	1985	0.24	-0.09	0.57
Unstandardized uncorrected CPUE	1986	0.25	-0.05	0.55
Unstandardized uncorrected CPUE	1987	0.23	-0.14	0.61
Unstandardized uncorrected CPUE	1988	0.20	-0.05	0.46
Unstandardized uncorrected CPUE	1989	0.23	-0.02	0.49
Unstandardized uncorrected CPUE	1990	0.21	0.02	0.39
Unstandardized uncorrected CPUE	1991	0.23	-0.02	0.48
Unstandardized uncorrected CPUE	1992	0.18	-0.08	0.43
Unstandardized uncorrected CPUE	1993	0.20	-0.03	0.43
Unstandardized uncorrected CPUE	1994	0.24	-0.03	0.51
Unstandardized uncorrected CPUE	1995	0.27	-0.03	0.57
Unstandardized uncorrected CPUE	1996	0.23	-0.03	0.49
Unstandardized uncorrected CPUE	1997	0.24	-0.05	0.52
Unstandardized uncorrected CPUE	1998	0.22	0.01	0.43
Unstandardized uncorrected CPUE	1999	0.22	-0.06	0.50
Unstandardized uncorrected CPUE	2000	0.16	0.00	0.31
Unstandardized uncorrected CPUE	2001	0.16	-0.03	0.35
Unstandardized uncorrected CPUE	2002	0.19	-0.03	0.40
Unstandardized uncorrected CPUE	2003	0.24	-0.01	0.50
Unstandardized uncorrected CPUE	2004	0.19	-0.01	0.39

Unstandardized uncorrected CPUE	2005	0.24	-0.11	0.58
Unstandardized uncorrected CPUE	2006	0.23	-0.09	0.56
Unstandardized uncorrected CPUE	2007	0.22	-0.04	0.48
Unstandardized uncorrected CPUE	2008	0.21	-0.09	0.52
Unstandardized uncorrected CPUE	2009	0.25	-0.02	0.52
Unstandardized uncorrected CPUE	2010	0.28	-0.01	0.56
Unstandardized uncorrected CPUE	2011	0.28	-0.01	0.56
Unstandardized uncorrected CPUE	2012	0.22	0.02	0.41
Unstandardized uncorrected CPUE	2013	0.24	-0.03	0.51
Unstandardized uncorrected CPUE	2014	0.21	-0.01	0.43
Unstandardized uncorrected CPUE	2015	0.22	-0.09	0.53
Unstandardized uncorrected CPUE	2016	0.19	0.02	0.37
Unstandardized uncorrected CPUE	2017	0.23	-0.07	0.53
Unstandardized uncorrected CPUE	2018	0.18	-0.03	0.39
Unstandardized uncorrected CPUE	2019	0.19	-0.05	0.43
Unstandardized corrected CPUE	1928	1.00	-0.25	2.25
Unstandardized corrected CPUE	1929	0.90	0.16	1.64
Unstandardized corrected CPUE	1930	0.58	0.13	1.02
Unstandardized corrected CPUE	1931	0.70	0.17	1.23
Unstandardized corrected CPUE	1932	0.65	0.18	1.12
Unstandardized corrected CPUE	1933	0.56	0.08	1.04
Unstandardized corrected CPUE	1934	0.43	0.05	0.81
Unstandardized corrected CPUE	1935	0.48	-0.02	0.97
Unstandardized corrected CPUE	1936	0.65	0.06	1.25
Unstandardized corrected CPUE	1937	0.68	0.10	1.26
Unstandardized corrected CPUE	1938	0.41	0.09	0.73
Unstandardized corrected CPUE	1939	0.84	-0.16	1.85
Unstandardized corrected CPUE	1940	0.64	0.08	1.19
Unstandardized corrected CPUE	1941	0.57	-0.07	1.21
Unstandardized corrected CPUE	1942	0.70	0.17	1.24
Unstandardized corrected CPUE	1943	0.56	0.15	0.98
Unstandardized corrected CPUE	1944	0.71	0.19	1.22
Unstandardized corrected CPUE	1945	0.96	0.24	1.68
Unstandardized corrected CPUE	1946	0.77	0.01	1.52
Unstandardized corrected CPUE	1947	0.65	0.10	1.20
Unstandardized corrected CPUE	1948	0.63	0.16	1.11
Unstandardized corrected CPUE	1949	0.81	0.21	1.42
Unstandardized corrected CPUE	1950	0.58	0.16	1.01
Unstandardized corrected CPUE	1951	0.58	0.18	0.98
Unstandardized corrected CPUE	1952	0.64	0.09	1.18
Unstandardized corrected CPUE	1953	0.60	0.07	1.13
Unstandardized corrected CPUE	1954	0.62	0.11	1.13
Unstandardized corrected CPUE	1955	0.47	-0.12	1.07
Unstandardized corrected CPUE	1956	0.86	-0.13	1.85
Unstandardized corrected CPUE	1957	0.37	-0.06	0.80
Unstandardized corrected CPUE	1958	0.44	0.05	0.83
Unstandardized corrected CPUE	1959	0.36	-0.05	0.76
Unstandardized corrected CPUE	1960	0.46	0.10	0.82

Unstandardized corrected CPUE	1961	0.36	0.00	0.71
Unstandardized corrected CPUE	1962	0.44	-0.11	0.99
Unstandardized corrected CPUE	1963	0.38	0.03	0.74
Unstandardized corrected CPUE	1964	0.39	-0.16	0.94
Unstandardized corrected CPUE	1965	0.26	-0.15	0.66
Unstandardized corrected CPUE	1966	0.34	0.01	0.67
Unstandardized corrected CPUE	1967	0.30	-0.32	0.91
Unstandardized corrected CPUE	1968	0.21	-0.04	0.46
Unstandardized corrected CPUE	1969	0.28	-0.01	0.57
Unstandardized corrected CPUE	1970	0.30	0.06	0.55
Unstandardized corrected CPUE	1971	0.16	0.02	0.31
Unstandardized corrected CPUE	1972	0.22	0.06	0.39
Unstandardized corrected CPUE	1973	0.26	-0.18	0.71
Unstandardized corrected CPUE	1974	0.21	-0.01	0.43
Unstandardized corrected CPUE	1975	0.24	0.02	0.45
Unstandardized corrected CPUE	1976	0.26	-0.11	0.62
Unstandardized corrected CPUE	1977	0.27	-0.08	0.62
Unstandardized corrected CPUE	1978	0.21	0.01	0.41
Unstandardized corrected CPUE	1979	0.21	0.00	0.42
Unstandardized corrected CPUE	1980	0.16	-0.05	0.36
Unstandardized corrected CPUE	1981	0.17	-0.04	0.37
Unstandardized corrected CPUE	1982	0.16	-0.10	0.43
Unstandardized corrected CPUE	1983	0.15	-0.06	0.36
Unstandardized corrected CPUE	1984	0.15	-0.09	0.39
Unstandardized corrected CPUE	1985	0.13	-0.05	0.31
Unstandardized corrected CPUE	1986	0.14	-0.03	0.30
Unstandardized corrected CPUE	1987	0.13	-0.08	0.33
Unstandardized corrected CPUE	1988	0.11	-0.03	0.25
Unstandardized corrected CPUE	1989	0.13	-0.01	0.27
Unstandardized corrected CPUE	1990	0.08	0.01	0.16
Unstandardized corrected CPUE	1991	0.09	-0.01	0.19
Unstandardized corrected CPUE	1992	0.07	-0.03	0.17
Unstandardized corrected CPUE	1993	0.08	-0.01	0.17
Unstandardized corrected CPUE	1994	0.10	-0.01	0.21
Unstandardized corrected CPUE	1995	0.11	-0.01	0.23
Unstandardized corrected CPUE	1996	0.09	-0.01	0.20
Unstandardized corrected CPUE	1997	0.10	-0.02	0.21
Unstandardized corrected CPUE	1998	0.09	0.00	0.17
Unstandardized corrected CPUE	1999	0.09	-0.02	0.20
Unstandardized corrected CPUE	2000	0.05	0.00	0.10
Unstandardized corrected CPUE	2001	0.05	-0.01	0.11
Unstandardized corrected CPUE	2002	0.06	-0.01	0.13
Unstandardized corrected CPUE	2003	0.08	0.00	0.16
Unstandardized corrected CPUE	2004	0.06	0.00	0.12
Unstandardized corrected CPUE	2005	0.07	-0.03	0.18
Unstandardized corrected CPUE	2006	0.07	-0.03	0.17
Unstandardized corrected CPUE	2007	0.07	-0.01	0.14
Unstandardized corrected CPUE	2008	0.06	-0.03	0.15

Unstandardized corrected CPUE	2009	0.07	-0.01	0.15
Unstandardized corrected CPUE	2010	0.08	0.00	0.15
Unstandardized corrected CPUE	2011	0.08	0.00	0.15
Unstandardized corrected CPUE	2012	0.06	0.01	0.11
Unstandardized corrected CPUE	2013	0.06	-0.01	0.14
Unstandardized corrected CPUE	2014	0.06	0.00	0.12
Unstandardized corrected CPUE	2015	0.06	-0.02	0.14
Unstandardized corrected CPUE	2016	0.05	0.01	0.10
Unstandardized corrected CPUE	2017	0.06	-0.02	0.14
Unstandardized corrected CPUE	2018	0.05	-0.01	0.10
Unstandardized corrected CPUE	2019	0.05	-0.01	0.11

Table S4. Technological data for traps used in the study. Measurements in centimeter. Trap type 1; cylindrical traps, 2; wooden traps, 3; wooden two-chamber traps and 4; synthetic two-chamber traps. Funnel is measured at the funnel eye (narrowest part of the funnel).

	Trap size			Frame		Funnel		
Trap type	Lenght of base	Max width	Max height	Frame/cover material	Net material	Eye material	Max width	Max height
1	98	39	39	wood/cotton	cotton	wood	8.5	8.5
1	86.5	41	41	wood /cotton	cotton	wood	8.5	8.5
1	86.5	38	38	wood /cotton	cotton	wood	10.0	9.5
1	86	41	41	wood /cotton	cotton	wood	8.0	9.0
1	89	41	41	wood /cotton	cotton	wood	8.5	8.5
1	82	41	41	wood /cotton	cotton	wood	9.0	8.0
1	83	41	41	wood /cotton	cotton	wood	8.0	8.0
1	84	41	41	wood /cotton	cotton	wood	8.0	8.0
1	88	39	39	wood /cotton	cotton	wood	8.5	8.5
1	83	39	39	wood /cotton	cotton	wood	8.5	7.0
1	84	37	37	wood /cotton	cotton	wood	8.0	7.5
1	86	41	41	wood /cotton	cotton	wood	8.5	8.5
1	84	40	40	wood /cotton	cotton	wood	9.0	8.0
1	88	37	37	wood /cotton	cotton	wood	9.0	8.5
1	84	41	41	wood /cotton	cotton	wood	8.5	9.0
2	80	42	26	wood	pvc	iron	10.0	10.0
2	80	42	26	wood	pvc	iron	10.0	10.0
2	80	42	26	wood	pvc	iron	10.0	10.0
2	80	42	26	wood	pvc	iron	10.0	10.0
2	80	42	26	wood	pvc	iron	10.0	10.0
2	80	42	26	wood	pvc	iron	10.0	10.0
2	80	42	26	wood	pvc	iron	10.0	10.0
2	80	42	26	wood	pvc	iron	10.0	10.5
2	80	42	26	wood	pvc	iron	10.0	11.0
3	84	39	31	wood	pvc	twine	12.0	14.0
3	84	39	31	wood	pvc	twine	13.0	14.5

3	84	39	31	wood	pvc	twine	13.0	16.0
3	84	39	31	wood	pvc	twine	13.5	14.0
3	84	39	31	wood	pvc	twine	13.0	15.5
3	84	39	31	wood	pvc	twine	14.5	16.0
3	84	39	31	wood	pvc	twine	13.5	15.0
3	86	40	32	wood	pvc	iron	11.5	12.0
3	86	39	32	wood	pvc	iron	12.0	12.0
3	86	39	32	wood	pvc	iron	13.0	12.5
4	92	40	45	steel/pvc	pvc	pvc	12.0	12.0
4	92	40	45	steel/pvc	pvc	pvc	12.0	12.0
4	92	40	45	steel/pvc	pvc	pvc	12.0	12.0
4	92	40	45	steel/pvc	pvc	pvc	12.0	12.0
4	92	40	45	steel/pvc	pvc	pvc	12.0	12.0
4	92	40	45	steel/pvc	pvc	pvc	12.0	12.0
4	92	40	45	steel/pvc	pvc	pvc	12.0	12.0
4	92	40	45	steel/pvc	pvc	pvc	12.0	12.0
4	92	40	45	steel/pvc	pvc	pvc	12.0	12.0
4	92	40	45	steel/pvc	pvc	pvc	12.0	12.0

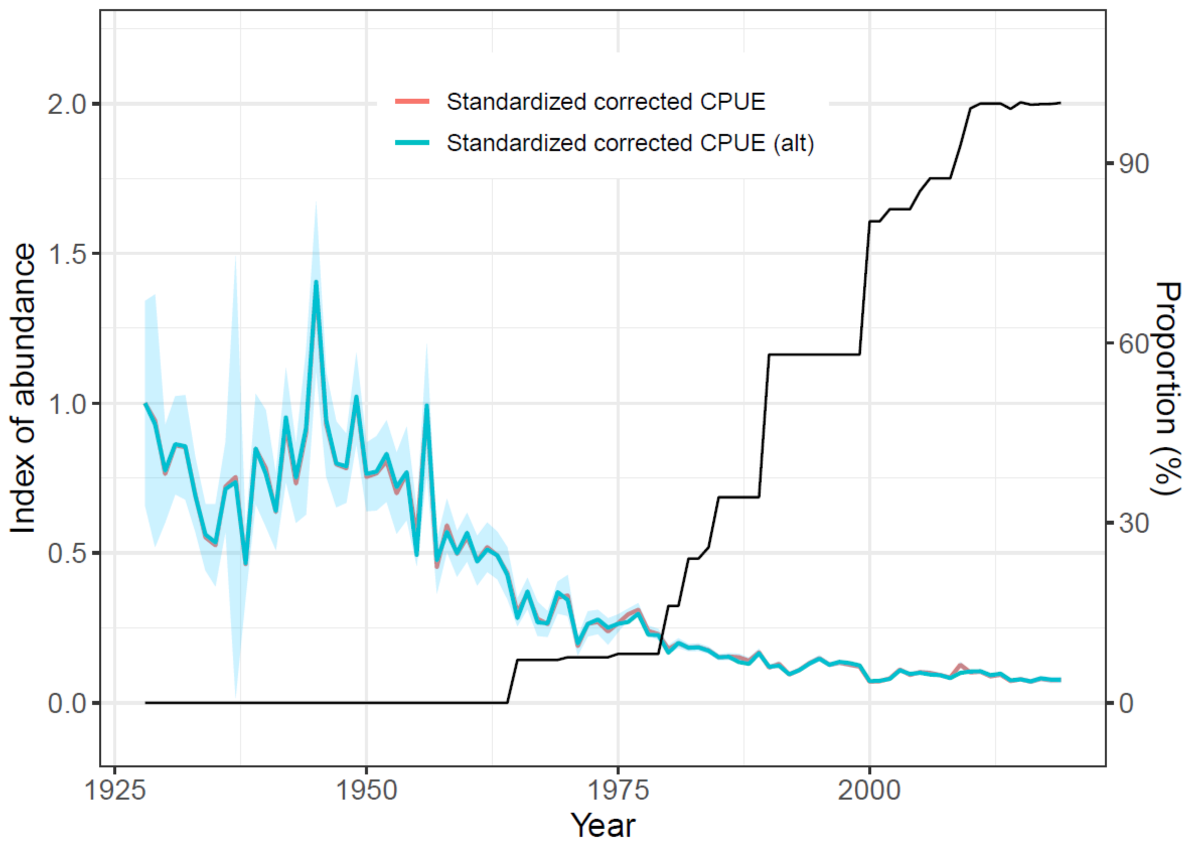

Figure S1. Lobster abundance indices (standardized and corrected for technological creep) based on the depletion model (red) and the alternate model based on GLMM (light blue)

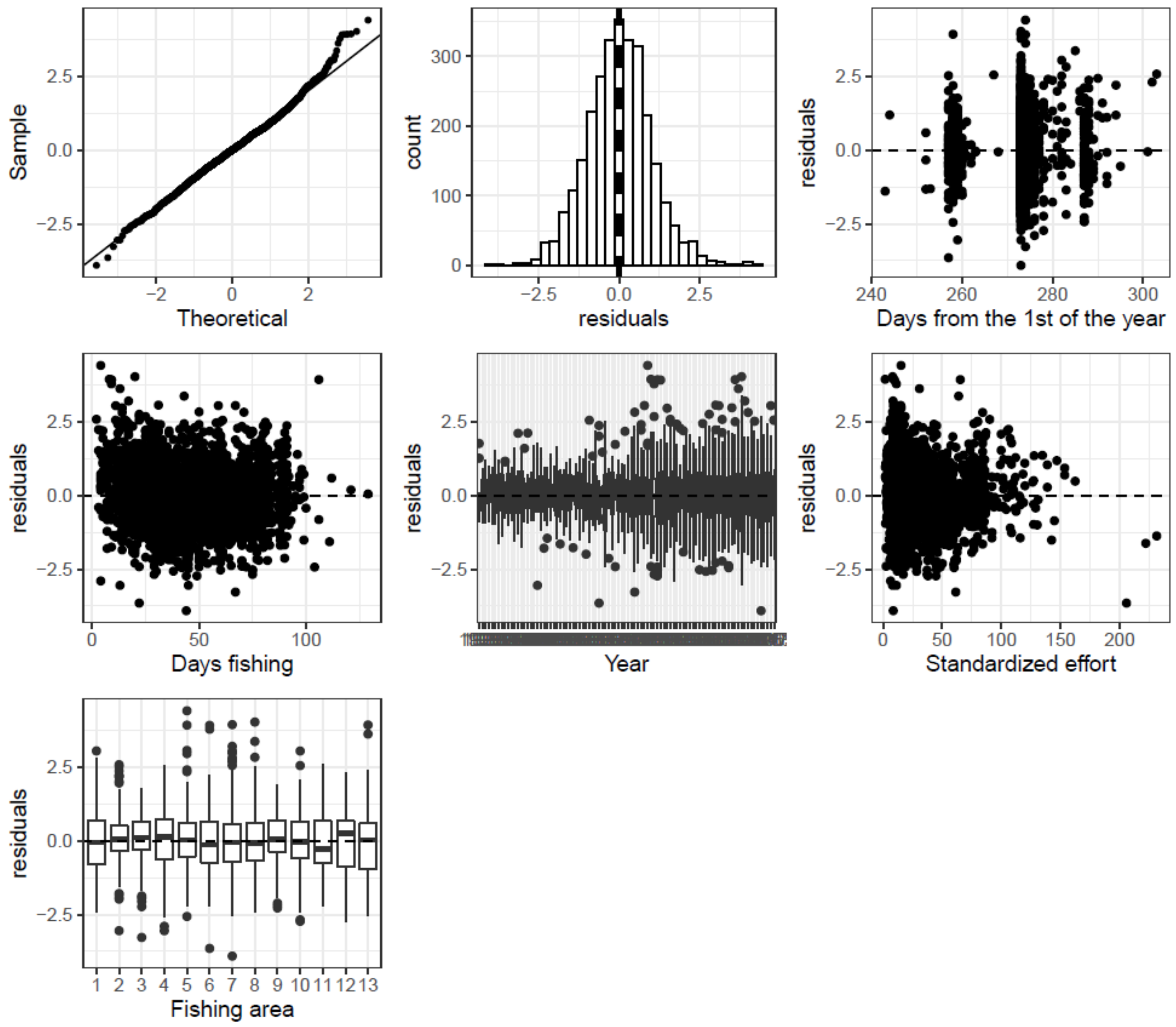

Figure S2. Residual diagnostic plots for the lobster depletion model. Diagnostics include residual Q-Q plot, histogram of standardized residual, and plots of residuals against covariates

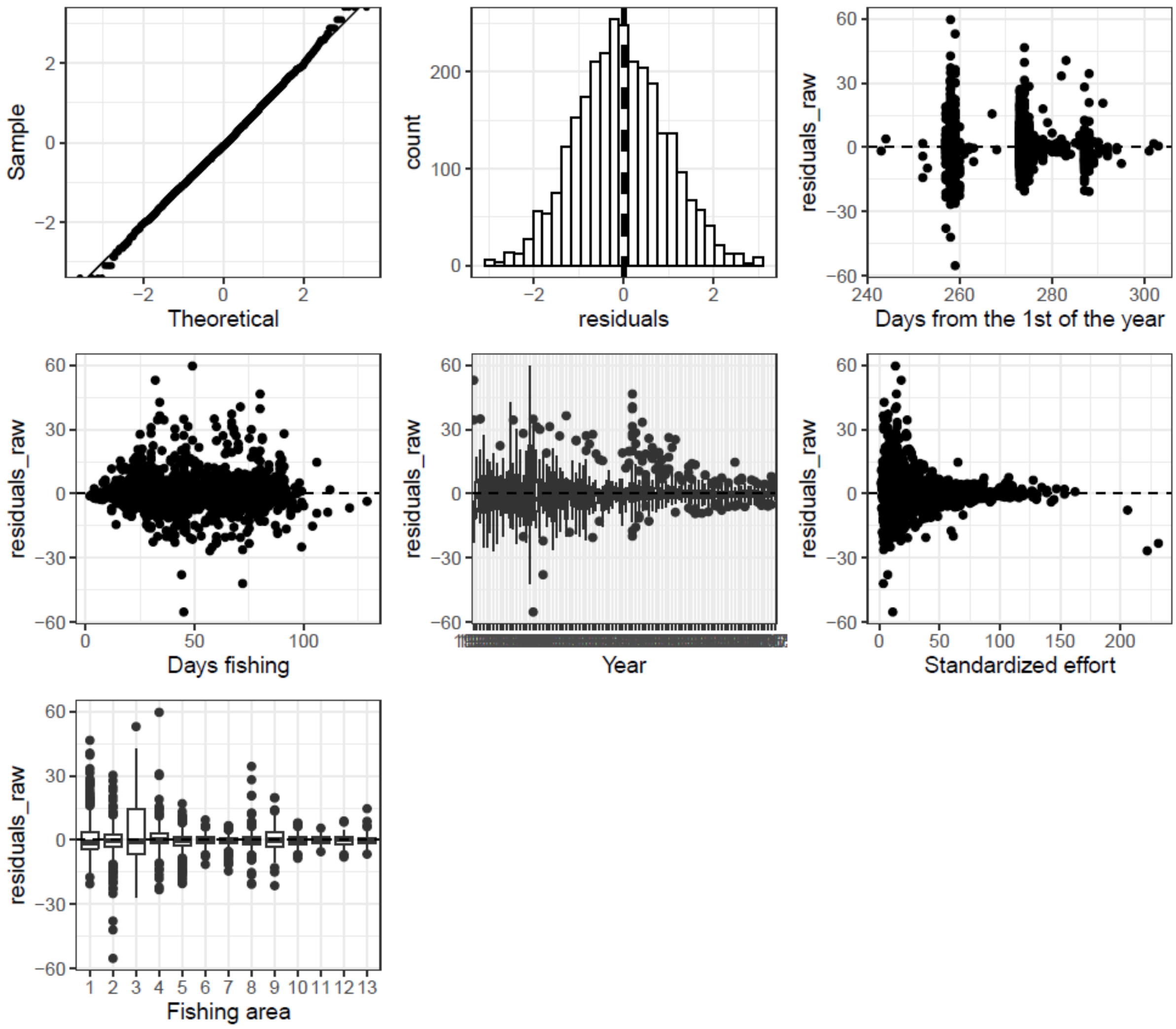

Figure S3. Residual diagnostic plots for the alternative lobster depletion model (fitted using glmmTMB). Diagnostics include residual Q-Q plot, histogram of standardized residual, and plots of residuals against covariates

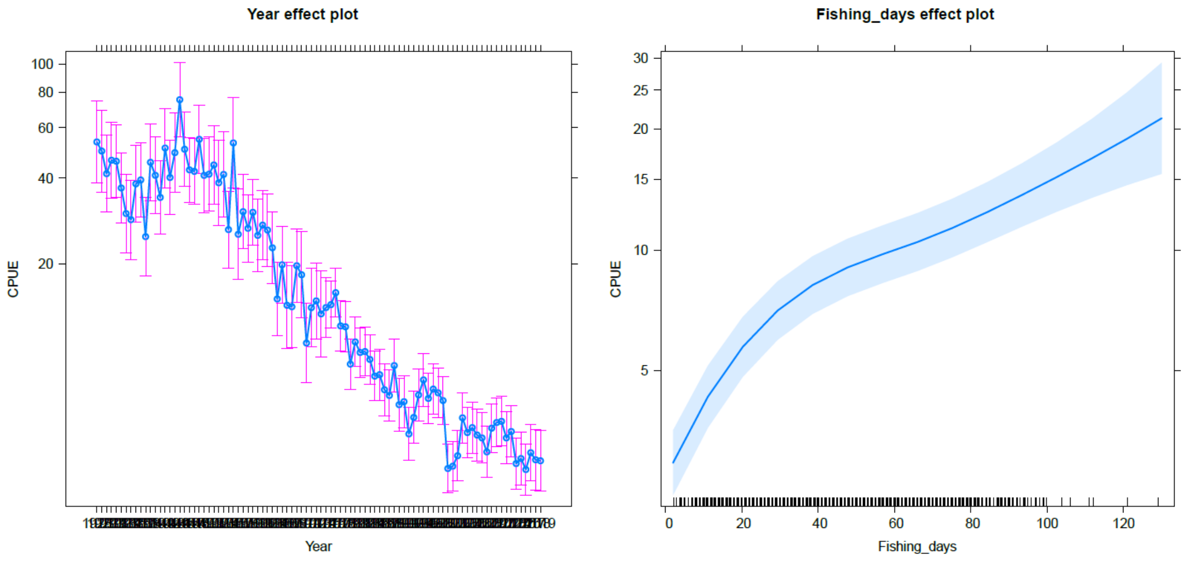

Figure S4. Estimated Year and Fishing days (i.e. season length) effect from the alternative lobster depletion model (fitted using glmmTMB).

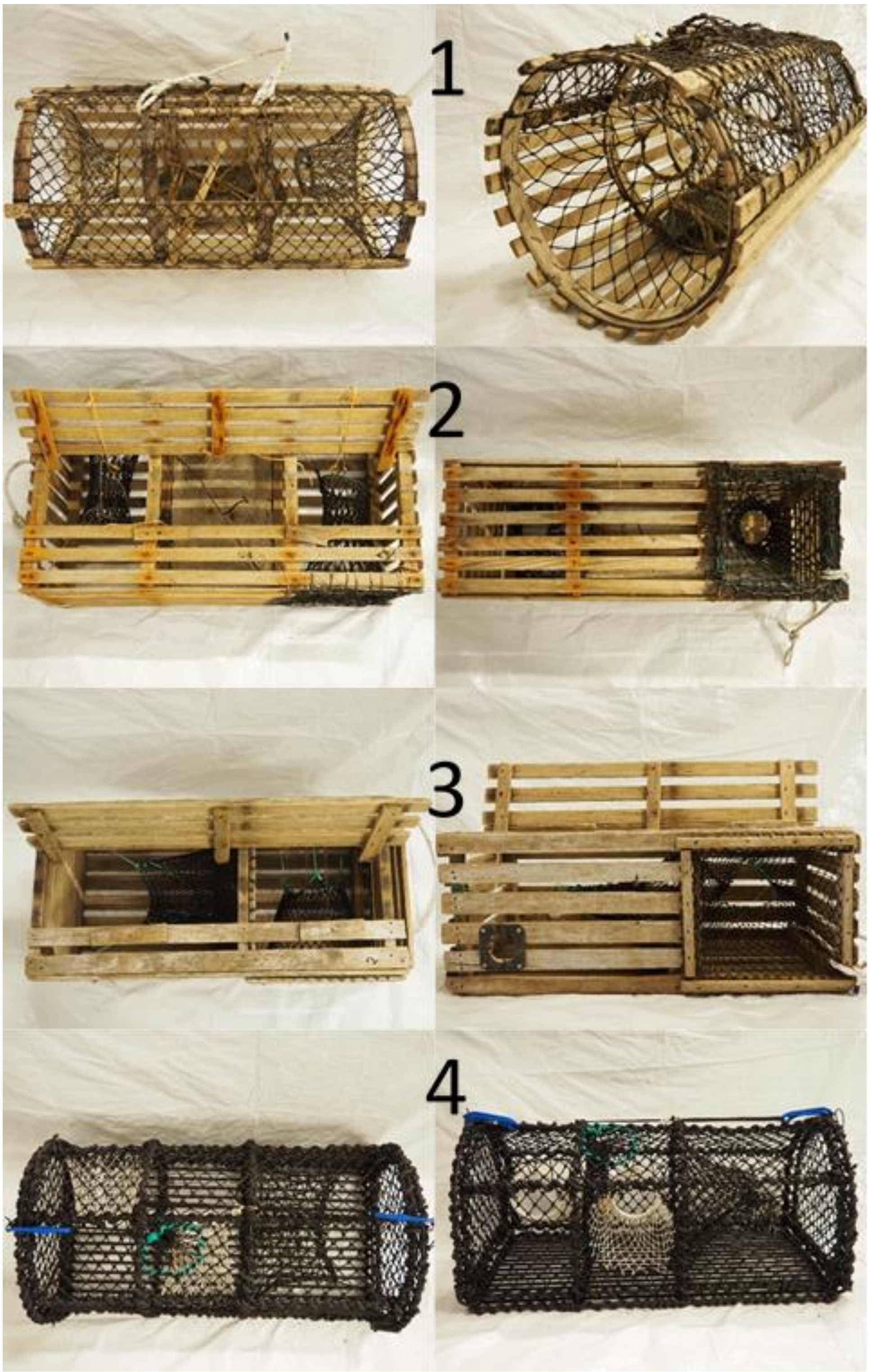

Figure S5. Traps identified as most common throughout the time series and used in the experimental test fishing. Trap type 1; cylindrical trap, 2; wooden trap, 3; wooden two-chamber trap and 4; synthetic two-chamber trap.

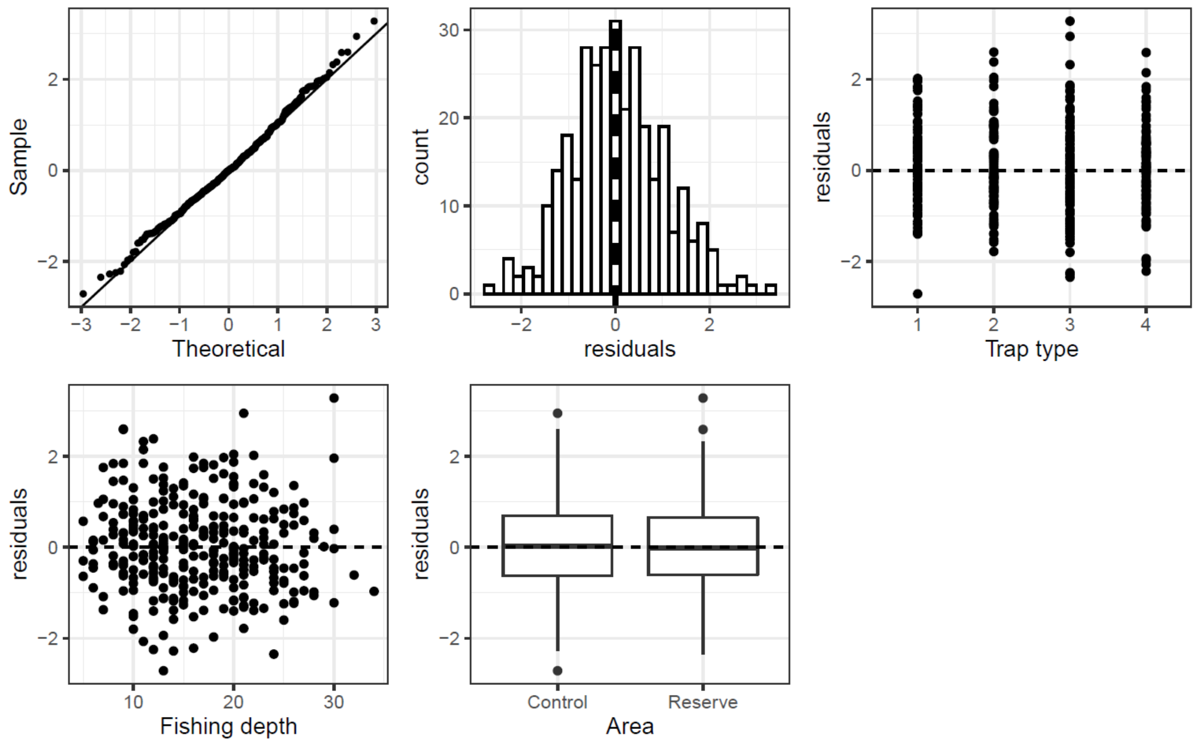

Figure S6. Residual diagnostic plots for the glmmTMB model to estimate lobster catchability by trap types. Diagnostics include residual Q-Q plot, histogram of standardized residual, and plots of residuals against covariates

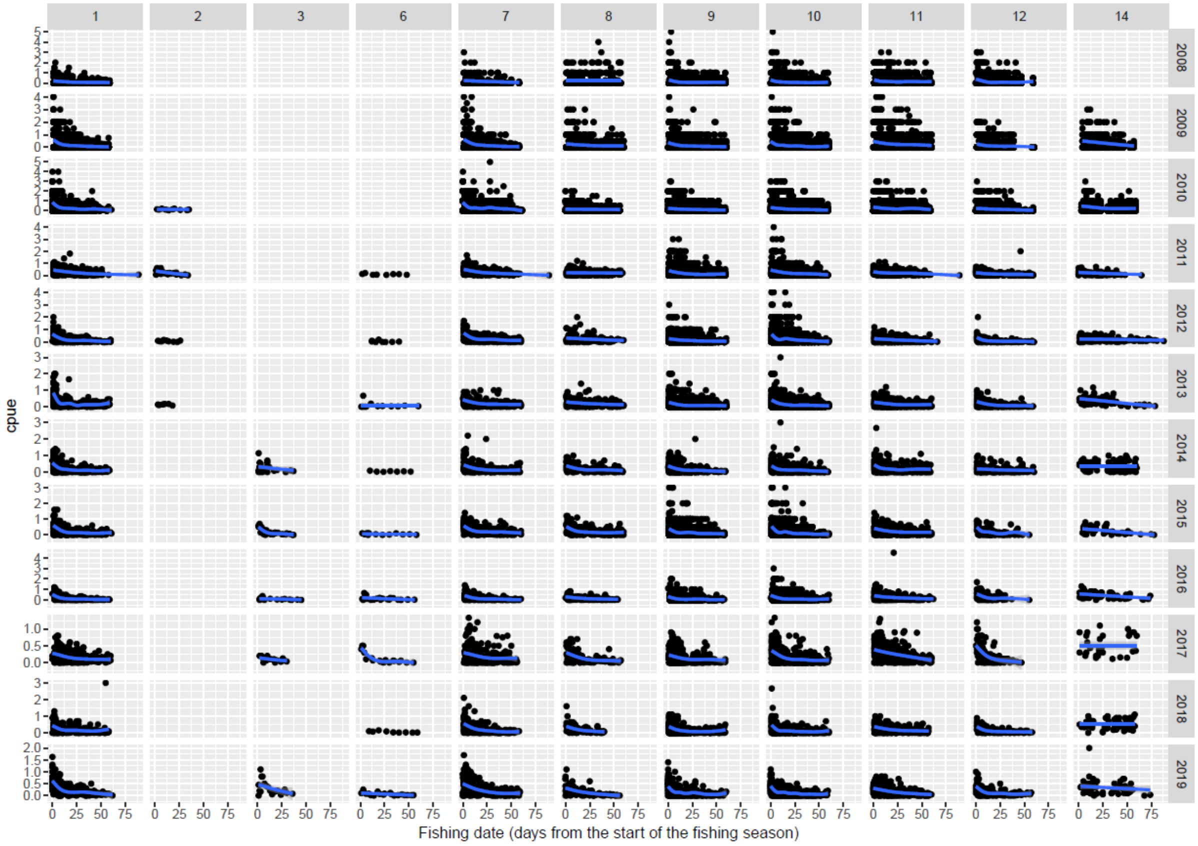

Figure S7. Lobster CPUE by area and year from the logbook data collected between 2008-2019 (whole season). Fishers reported number of traps used, number of landed lobsters and time fishing (start and end date). Mean annual reports; 48.3. The black dots represent individual observations and the blue line is the smoothing spline fitted to the observations. Numbers given in the upper row are different areas along the southern Norwegian coast. A map showing the areas are given in Figure S4.

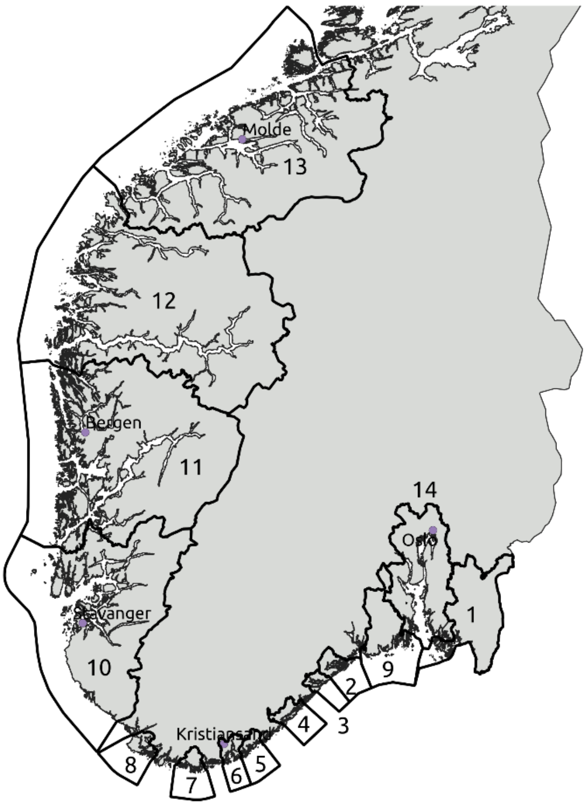

Figure S8. Map of southern Norway showing the sampling areas of CPUE data reported by fishers from 1928 to 2019. Numbers given in the map is the code for the given area and is found in Figure S7.

Equation S1. Mathematical derivation of the depletion model

$$\int_{t=start_i}^{t=end_i} \alpha e^{-\beta t}\, dt = \frac{\alpha}{\beta} e^{-\beta start_i} - \frac{\alpha}{\beta} e^{-\beta end_i}$$

$$= \frac{\alpha}{\beta}\left(e^{-\beta start_i} - e^{-\beta end_i}\right)$$

$$CPUE_i = \frac{1}{end_i - start_i}\frac{\alpha}{\beta}\left(e^{-\beta start_i} - e^{-\beta end_i}\right)$$
